# Cyclooxygenase-2 in adipose tissue macrophages limits adipose tissue dysfunction in obese mice

**DOI:** 10.1172/JCI152391

**Published:** 2022-05-02

**Authors:** Yu Pan, Shirong Cao, Jiaqi Tang, Juan P. Arroyo, Andrew S. Terker, Yinqiu Wang, Aolei Niu, Xiaofeng Fan, Suwan Wang, Yahua Zhang, Ming Jiang, David H. Wasserman, Ming-Zhi Zhang, Raymond C. Harris

**Affiliations:** 1 Division of Nephrology and Hypertension, Department of Medicine and; 2 Vanderbilt Center for Kidney Disease, Vanderbilt University Medical Center, Nashville, Tennessee, USA.; 3 Division of Nephrology, Shanghai Ninth People’s Hospital, Shanghai Jiao Tong University School of Medicine, Shanghai, China.; 4 Veterans Affairs, Nashville, Tennessee, USA.; 5 Department of Molecular Physiology and Biophysics, Vanderbilt University School of Medicine, Nashville, Tennessee, USA.

**Keywords:** Inflammation, Metabolism, Adipose tissue, Eicosanoids, Obesity

## Abstract

Obesity-associated complications are causing increasing morbidity and mortality worldwide. Expansion of adipose tissue in obesity leads to a state of low-grade chronic inflammation and dysregulated metabolism, resulting in insulin resistance and metabolic syndrome. Adipose tissue macrophages (ATMs) accumulate in obesity and are a source of proinflammatory cytokines that further aggravate adipocyte dysfunction. Macrophages are rich sources of cyclooxygenase (COX), the rate limiting enzyme for prostaglandin E2 (PGE2) production. When mice were fed a high-fat diet (HFD), ATMs increased expression of COX-2. Selective myeloid cell COX-2 deletion resulted in increased monocyte recruitment and proliferation of ATMs, leading to increased proinflammatory ATMs with decreased phagocytic ability. There were increased weight gain and adiposity, decreased peripheral insulin sensitivity and glucose utilization, increased adipose tissue inflammation and fibrosis, and abnormal adipose tissue angiogenesis. HFD pair-feeding led to similar increases in body weight, but mice with selective myeloid cell COX-2 still exhibited decreased peripheral insulin sensitivity and glucose utilization. Selective myeloid deletion of the macrophage PGE2 receptor subtype, EP4, produced a similar phenotype, and a selective EP4 agonist ameliorated the metabolic abnormalities seen with ATM COX-2 deletion. Therefore, these studies demonstrated that an ATM COX-2/PGE2/EP4 axis plays an important role in inhibiting adipose tissue dysfunction.

## Graphical abstract

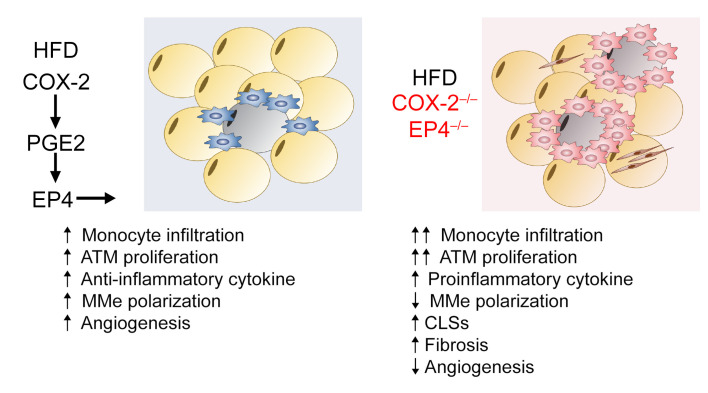

## Introduction

There are currently about 2 billion people globally who are obese or overweight, leading to health complications, type 2 diabetes, cardiovascular disease, and liver disease. The expansion of adipose tissue in obesity leads to a state of low-grade chronic inflammation and dysregulated metabolism, resulting in insulin resistance and development of metabolic syndrome.

Three major abnormalities promote dysfunctional adipose tissue: persistent inflammation, inappropriate extracellular matrix deposition, and abnormalities in angiogenesis (1, 2). Under these conditions, there is an accumulation in adipose tissue of immune cells, especially adipose tissue macrophages (ATMs), that are the source of proinflammatory cytokines that further aggravate adipocyte dysfunction (3–5). However, mechanisms underlying the increases in ATMs and their proinflammatory phenotype are still not fully understood.

Macrophages are rich sources of cyclooxygenase (COX), and the most prevalent prostanoid produced by macrophages is prostaglandin E2 (PGE2). PGE2 can be either proinflammatory or antiinflammatory depending on the context. The cellular responses mediated by PGE2 result from activation of 4 specific membrane-associated G protein–coupled receptors, EP1 through EP4 (6–9). EP4, which is primarily coupled to G_s_, is the predominant PGE2 receptor in macrophages (10).

Two isoforms of COX exist in mammals, constitutive COX-1 and inducible COX-2. Although both COX isoforms are expressed in macrophages, COX-2 expression increases in response to inflammation (6, 11). NSAIDs inhibit PGE2 production by nonselective inhibition of COX enzymatic activity.

Recent studies have shown that obesity leads to a proinflammatory and metabolically activated adipose tissue macrophage phenotype (MMe), which is mechanistically and functionally distinct from the classic proinflammatory M1 phenotype and driven in part by saturated fatty acids (such as palmitic acid) released by insulin-resistant adipocytes (12–14). MMe macrophages express mediators of lipid metabolism, such as perilipin-1 and CD36, as well as proinflammatory cytokines, but they also play an essential role in the phagocytic clearance of apoptotic adipocytes during diet-induced obesity (DIO). Dysregulated macrophage MMe polarization not only impairs clearance of apoptotic adipocytes but also accelerates the development of insulin resistance. The ATM COX-2 response to DIO has not been previously investigated. Therefore, the current studies examined the role of COX-2 and the PGE2 receptor, EP4, in mediating ATM recruitment, proliferation, and polarization and their role in adipose inflammation associated with obesity and found that COX-2 in ATMs limited adipose tissue dysfunction in obesity.

## Results

### ATM COX-2 expression increased in response to a high-fat diet.

**Figure 1:**
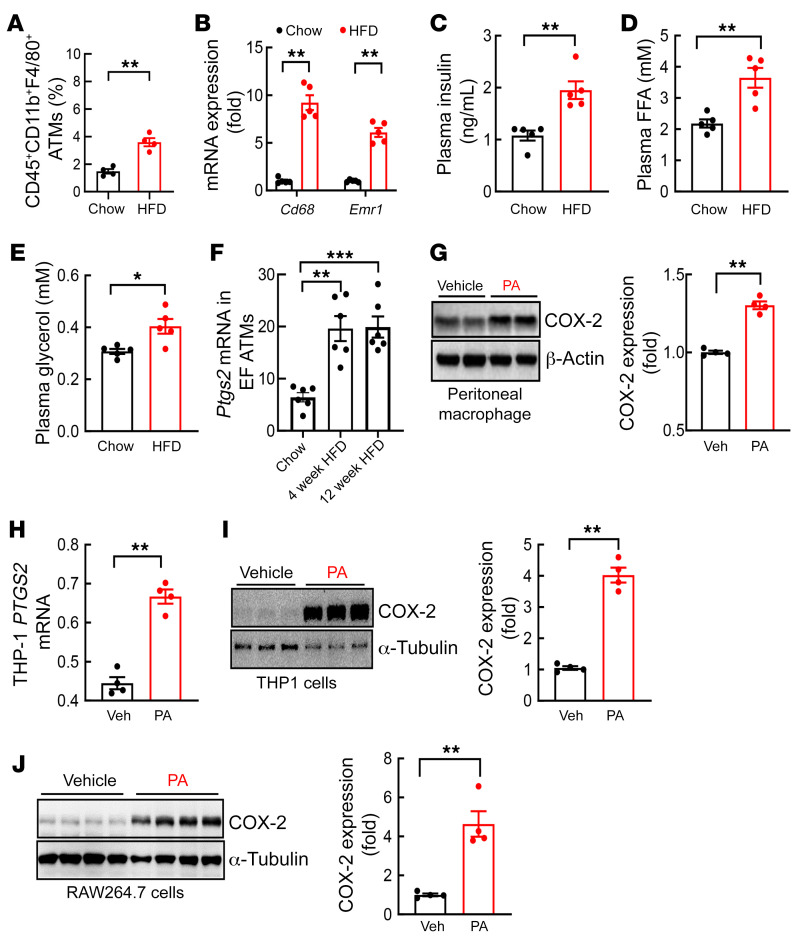
COX-2 expression in adipose tissue macrophages from epididymal fat increased in the early phase of DIO. Male FVB mice were fed an HFD for 4 or 12 weeks. (**A**) Flow cytometry gating on the SVF indicated that the HFD led to increased EF CD45^+^CD11b^+^F4/80^+^ ATMs (*n =* 4). (**B**–**E**) Four weeks of the HFD led to increases in mRNA levels of both EF *Cd68* and *Emr1* (**B**), plasma levels of insulin (**C**), FFA (**D**), and glycerol (**E**) (*n =* 5). (**F**) Isolated EF ATM *Ptgs2* mRNA levels were increased in mice with the HFD for 4 or 12 weeks (*n =* 6). (**G**–**J**) PA stimulated COX-2 expression in isolated mouse peritoneal macrophages (**G**), in the human macrophage-like THP1 cells (**H** and **I**), and in the mouse macrophage-like RAW264.7 cells (**J**) (*n =* 3–4 independent repeats). Data are mean ± SEM. *P < 0.05, **P < 0.01, ***P < 0.001, analyzed using 2-tailed Student’s *t* test for **A**–**E** and **G**–**J** and 2-way ANOVA followed by Bonferroni’s post hoc test for **F**. EF, epididymal fat; FFA, free fatty acid; PA, palmitic acid; SVF, stromal vascular fraction.

DIO is characterized by increased ATMs and increased lipolysis and release of saturated free fatty acid (FFA) and glycerol secondary to insulin insensitivity (15). WT mice were fed normal chow or a high-fat diet (HFD; 36% fat accounting for 60% of calories) for 4 weeks. As expected, the HFD led to increased body weight and fasting blood glucose (Supplemental Figure 1; supplemental material available online with this article; https://doi.org/10.1172/JCI152391DS1). Flow cytometry confirmed increased ATMs (CD45^+^CD11b^+^F4/80^+^) in epididymal tissue after 4 weeks of the HFD (Figure 1A). Four weeks of the HFD also led to increased epididymal adipose tissue mRNA expression of *Emr1* (F4/80) and *Cd68*, two markers of ATMs (Figure 1B), as well as increased plasma insulin, FFA, and glycerol concentrations (Figure 1, C–E).

ATMs are a major source of proinflammatory cytokines that promote insulin resistance and metabolic syndrome in DIO. COX-2 is highly expressed in macrophages. We compared COX-2 expression in ATMs isolated from epididymal adipose tissue from mice on normal chow or the HFD. *Ptgs2* (COX-2) mRNA levels were markedly higher in ATMs isolated from epididymal tissue from WT mice fed the HFD for 4 weeks or 12 weeks (Figure 1F). Palmitic acid, the major FFA released by adipocytes, increased COX-2 expression in mouse peritoneal macrophages (PMs; Figure 1G) and human (THP1) (Figure 1, H and I) and mouse (RAW264.7; Figure 1J) macrophage-like cell lines.

### COX-2 expression was effectively deleted in ATMs from CD11b-Cre COX-2^fl/fl^ mice.

**Figure 2:**
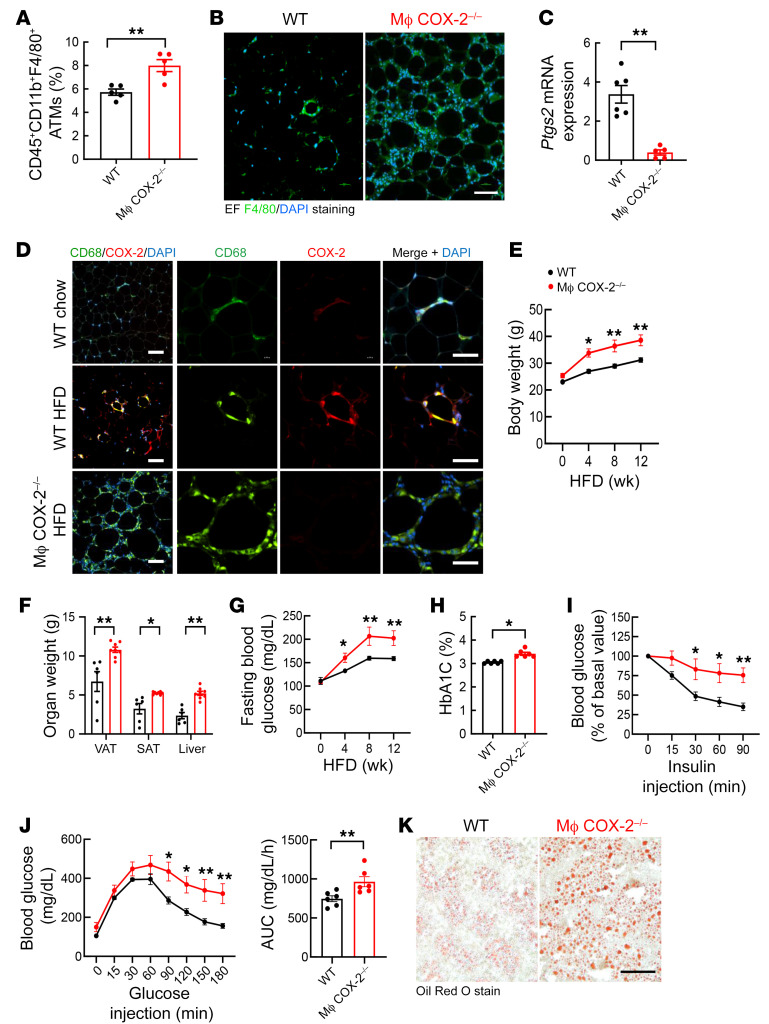
Myeloid COX-2^–/–^ mice had more significant metabolic abnormalities in DIO. WT (COX-2^fl/fl^) mice and myeloid COX-2^–/–^ (CD11b-Cre COX-2^fl/fl^) mice were fed the HFD for 12 weeks. (**A**) Flow cytometry gating on the SVF indicated greater increases in EF ATMs in myeloid COX-2^–/–^ mice (*n =* 5). (**B** and **C**) Myeloid COX-2^–/–^ mice had more EF F4/80-positive ATMs, (**B**) but lower *Ptgs2* mRNA levels (**C**), compared with WT mice (*n =* 5–6). Scale bar: 100 μm. (**D**) Representative images showed that COX-2 was expressed in many ATMs in crown-like structures in the HFD-treated WT mice but was minimally expressed in WT mice on normal chow and was undetectable in the HFD-treated myeloid COX-2^–/–^ mice. Scale bars: 100 μm (left) and 50 μm (right). (**E** and **F**) Myeloid COX-2^–/–^ mice had greater increases in body weight (*n =* 10) (**E**) and fat and liver masses (*n =* 6–8) (**F**). (**G**–**J**) Myeloid COX-2^–/–^ mice had greater increases in fasting blood glucose (*n =* 8) (**G**) and HbA1c (*n =* 6) (**H**) and decreased insulin tolerance (*n =* 8) (**I**) and glucose tolerance (*n =* 8) (**J**). (**K**) Representative images showed more severe liver steatosis in myeloid COX-2^–/–^ mice. Scale bar: 100 μm. Data are mean ± SEM. *P < 0.05, **P < 0.01, analyzed using 2-way ANOVA followed by Bonferroni’s post hoc test for **F**, 2-tailed Student’s *t* test for **A**, **C**, **F**, and **H**, 2-way ANOVA followed by Tukey’s post hoc test for **E**, **G**, and **I**, 2-tailed Student’s *t* test and 2-way ANOVA followed by Tukey’s post hoc test for **J**. EF, epididymal fat; SVF, stromal vascular fraction.

To investigate the potential role of myeloid COX-2 expression in development of DIO and insulin resistance, we crossed COX-2^fl/fl^ mice with CD11b-Cre mice (CD11b-Cre COX-2^fl/fl^, macrophage COX-2^–/–^) and utilized COX-2^fl/fl^ mice as WT controls. After 12 weeks of the HFD, flow cytometry indicated greater increases in ATMs in epididymal fat (EF) in macrophage COX-2^–/–^ mice than WT mice (Figure 2A). Immunoreactivity of F4/80, the macrophage marker, was increased in nonadipose cells in EF in macrophage COX-2^–/–^ mice compared with WT mice with the HFD (Figure 2B). EF *Ptgs2* mRNA levels were 10-fold lower in macrophage COX-2^–/–^ mice than WT mice (2.99 ± 0.29 vs. 0.30 ± 0.06 for WT, *P* < 0.01, *n =* 6) (Figure 2C). COX-2 protein expression was minimal in EF ATMs in WT mice with normal chow but was evident in ATMs in the crown-like structures (CLSs) in WT mice on the HFD for 12 weeks, while COX-2 was not detectable in EF ATMs in CLSs in macrophage COX-2^–/–^ mice on the HFD (Figure 2D). These findings confirmed increased COX-2 expression in ATMs in DIO and effective deletion in macrophage COX-2^–/–^ mice.

### Myeloid COX-2 deletion led to greater insulin resistance and metabolic alterations in DIO.

On the normal chow diet (4.5% fat for 13% of calories), there were no differences in body weights, fasting blood glucose, HbA1c, or glucose or insulin tolerance tests between WT and macrophage COX-2^–/–^ mice at either 20 or 40 weeks of age (Supplemental Figure 2). In contrast, on the HFD, body weight increased significantly more in macrophage COX-2^–/–^ mice (Figure 2E). Increases in subcutaneous adipose tissue (SAT) and visceral adipose tissue (VAT) mass and liver mass were the primary contributors to the increased body weight in macrophage COX-2^–/–^ mice (Figure 2F).

Fasting blood sugars were significantly increased in macrophage COX-2^–/–^ mice compared with WT mice by 4 weeks of the HFD and continued to be significantly increased throughout the subsequent 8 weeks (Figure 2G). HbA1c levels measured at 12 weeks on the HFD were also significantly higher in macrophage COX-2^–/–^ mice (Figure 2H). The macrophage COX-2^–/–^ mice had both abnormal insulin tolerance and glucose tolerance tests and more severe liver steatosis compared with WT mice after 12 weeks on the HFD (Figure 2, I–K).

Because we were able to detect increased COX-2 expression in ATMs by 4 weeks after initiation of the HFD, we also investigated earlier consequences and confirmed that after only 4 weeks of the HFD, the macrophage COX-2^–/–^ mice had abnormal glucose tolerance and insulin tolerance compared with WT mice (Supplemental Figure 3, A–C). Even a medium-fat diet (11% fat accounting for 25% of calories) for 4 weeks induced abnormal glucose tolerance and insulin tolerance in macrophage COX-2^–/–^ mice compared with WT mice (Supplemental Figure 3, D–F). We also treated COX-2^fl/fl^ mice, littermate CD11b-Cre mice, and CD11b-Cre COX-2^fl/fl^ mice with the HFD for 4 weeks. COX-2^fl/fl^ mice and CD11b-Cre mice had comparable body weight, blood glucose, glucose tolerance test, and insulin tolerance test at baseline and after the HFD for 4 weeks (Supplemental Figure 3, G–J). Of note, there were no differences among the groups when mice were fed a chow diet (data not shown).

We noted that macrophage COX-2^–/–^ mice had significantly greater food intake measured at 5 or 9 weeks after initiation of the HFD (Supplemental Figure 4A). Further analysis showed that the circadian rhythm of food intake was similar between WT and macrophage COX-2^–/–^ mice either on a chow diet or an HFD (Supplemental Figure 4, B and C). Given that body weight gain and increased adiposity can contribute to abnormal glucose tolerance and insulin tolerance tests, we used HFD pair-feeding to investigate further the potential role of ATM COX-2 deletion in the observed metabolic disturbances. Pair-feeding of the HFD for 4 weeks led to similar increases in body weight in WT and macrophage COX-2^–/–^ mice. However, the macrophage COX-2^–/–^ mice still exhibited increased fasting blood glucose and abnormal glucose tolerance tests and insulin tolerance tests compared with WT mice (Supplemental Figure 4, D–G).

**Figure 3:**
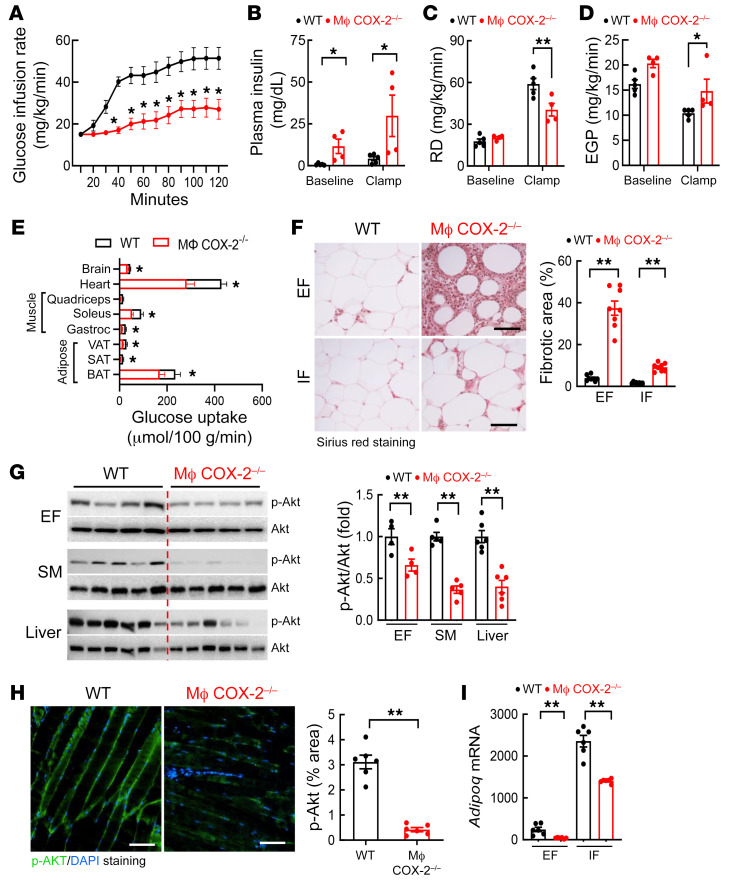
Myeloid COX-2^–/–^ mice had decreased insulin sensitivity in insulin-sensitive tissues after high-fat feeding. WT and myeloid COX-2^–/–^ mice were on the HFD for 11 weeks. (**A**) Hyperinsulinemic-euglycemic clamps determined more severe insulin resistance in myeloid COX-2^–/–^ mice, as less glucose infusion was needed to maintain a constant blood glucose (*n =* 4). (**B**–**D**) Myeloid COX-2^–/–^ mice had increased plasma insulin levels at baseline and during clamp periods (**B**), decreased rates of glucose disappearance (RD) (**C**), and increased endogenous glucose production (EGP) (**D**) (*n =* 4 and 5). (**E**) Myeloid COX-2^–/–^ mice had decreased glucose uptake, a marker of insulin resistance in adipose tissues (BAT, SAT, VAT), SM (gastrocnemius and soleus), and heart and brain (*n =* 4 and 5). (**F**) Picrosirius red staining indicated more fibrosis in EF and IF in myeloid COX-2^–/–^ mice than WT mice (*n =* 8). Scale bars: 100 μm. (**G**) Myeloid COX-2^–/–^ mice had decreased insulin-stimulated p-Akt in EF, SM, and liver, an indication of increased insulin resistance (*n =* 4–6). (**H**) Quantitative p-Akt immunofluorescent staining showed insulin insensitivity in SM in myeloid COX-2^–/–^ mice (*n =* 6). Scale bars: 100 μm. (**I**) Myeloid COX-2^–/–^ mice had lower *Adipoq* mRNA levels in EF and IF (*n =* 6). Data are mean ± SEM. *P < 0.05, **P < 0.01, analyzed using 2-way ANOVA followed by Tukey’s post hoc test for **A**, 2-way ANOVA followed by Bonferroni’s post hoc test for **B**–**D**, **F**, and **I**, and 2-tailed Student’s *t* test for **E**, **G**, and **H**. Brown, subcutaneous, and visceral adipose tissue (BAT, SAT, and VAT); EF, epididymal fat; IF inguinal fat; SM, skeletal muscle.

In hyperinsulinemic-euglycemic clamps of 5-hour fasted mice after 12 weeks on the HFD, macrophage COX-2^–/–^ mice required significantly less glucose administration in order to maintain a constant blood glucose throughout the course of the study (Figure 3A and Supplemental Figure 5). Macrophage COX-2^–/–^ mice had significantly higher plasma insulin levels at baseline and during the clamp (Figure 3B). Macrophage COX-2^–/–^ mice also exhibited a decreased rate of glucose disappearance (RD; Figure 3C), consistent with higher insulin concentrations needed for tissue glucose utilization as well as increased endogenous hepatic glucose production (Figure 3D). Macrophage COX-2^–/–^ mice had significantly less glucose uptake into SAT, VAT, and brown adipose tissue (BAT) (Figure 3E). There was also significantly less glucose uptake into skeletal muscles (soleus and gastrocnemius) as well as into the heart and brain (Figure 3E). After 12 weeks on the HFD, macrophage COX-2^–/–^ mice had significantly more fibrosis in EF (percentage fibrotic area: 37.44% ± 3.41% vs. 3.97% ± 0.48%, *P* < 0.01, *n =* 8) as well as in inguinal fat (IF) (Figure 3F), with less fibrosis in IF than in EF in both WT mice and macrophage COX-2^–/–^ mice. Decreased insulin-activated p-Akt levels in the adipose tissue, skeletal muscle, and liver further confirmed insulin insensitivity in macrophage COX-2^–/–^ mice (Figure 3, G and H). EF mRNA levels of *Adipoq* were more than 6-fold lower in macrophage COX-2^–/–^ mice than WT mice 12 weeks after the HFD (39.55 ± 5.85 vs. 249.35 ± 70.53, *P* < 0.01, *n =* 6) (Figure 3I). The *Adipoq* mRNA levels in IF were approximately 10 times higher than that in EF in WT mice and were only decreased by about 40% in macrophage COX-2^–/–^ mice (1421.12 ± 28.63 vs. 2323.68 ± 242.46, *P* < 0.01, *n =* 6) (Figure 3I).

### COX-2 deletion in adipocytes had minimal effects on development of insulin resistance and metabolic syndrome in DIO.

Given that adipose tissues are insulin sensitive, we also generated mice with selective COX-2 deletion in adipocytes (Adipoq-Cre COX-2^fl/fl^, adipocyte COX-2^–/–^) and COX-2^fl/fl^ (WT) mice and fed them the HFD for 16 weeks. Selective COX-2 deletion in adipocytes was confirmed (Supplemental Figure 6A). In contrast to macrophage COX-2^–/–^ mice, body weights, fasting blood glucose levels, and insulin tolerance tests were comparable between WT and adipocyte COX-2^–/–^ mice (Supplemental Figure 6, B–D).

### Myeloid COX-2 deletion induced adipose tissue dysfunction.

**Figure 4:**
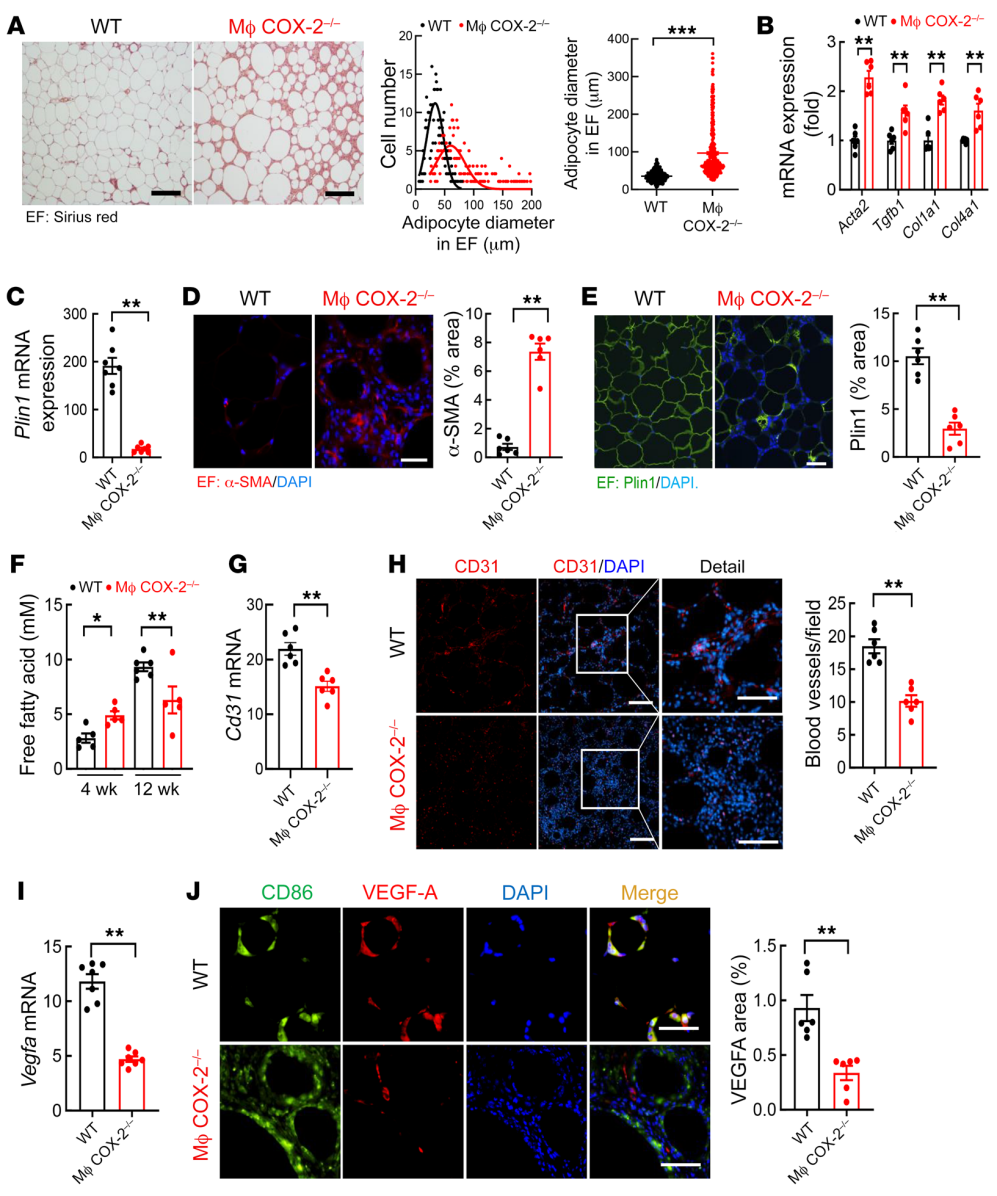
Myeloid COX-2^–/–^ mice had greater adipocyte hypertrophy, fat tissue fibrosis, and vascular rarefaction. WT and myeloid COX-2^–/–^ mice were on the HFD for 12 weeks. (**A**) Myeloid COX-2^–/–^ mice had larger adipocytes in EF (*n =* 390, 65 adipocytes measured from each of 6 mice in each group). Scale bars: 200 μm. (**B**–**E**) Myeloid COX-2^–/–^ mice had increased EF mRNA expression of profibrotic and fibrotic components, including *Acta2*, *Tgfb1*, *Col1a1*, and *Col4a1* (**B**), and increased α-SMA protein expression (**D**), but decreased EF expression of Plin1 mRNA (**C**) and protein (**E**) (*n =* 6 and 7). Scale bars: 100 μm. (**F**) Myeloid COX-2^–/–^ mice had higher plasma FFA levels at 4 weeks but lower FFA levels at 12 weeks after the HFD (*n =* 5 and 6). (**G** and **H**) Myeloid COX-2^–/–^ mice had decreased EF blood vessel density, as indicated by decreased *Cd31* mRNA expression (**G**) and quantitative CD31 immunofluorescent staining for blood vessels (**H**) (*n =* 6). Scale bars: 100 μm (left) and 50 μm (right). (**I**) Myeloid COX-2^–/–^ mice had decreased EF *Vegfa* (*n =* 7). (**J**) VEGF-A was primarily localized to ATMs in EF and its expression was markedly decreased in myeloid COX-2^–/–^ mice (*n =* 6). Scale bars: 50 μm. Data are mean ± SEM. *P < 0.05, **P < 0.01, ***P < 0.001, analyzed using 2-tailed Student’s *t* test for **A**–**E** and **G**–**J** and 2-way ANOVA followed by Bonferroni’s post hoc test for **F**. EF, epididymal fat.

Both increased adipocyte size (hypertrophy) and inappropriate extracellular matrix remodeling contribute to the pathogenesis of dysfunctional adipose tissue in obesity (1, 16). Adipocytes in both EF and IF were significantly larger in macrophage COX-2^–/–^ mice, as indicated by increased average adipocyte diameters (Figure 4A and Supplemental Figure 7A). Macrophage COX-2^–/–^ mice had significantly increased EF mRNA expression of *Acta2*, *Tgfb1*, *Co1la1*, and *Col4a1* (Figure 4B) as well as markedly decreased EF *Plin1* mRNA levels (191.4 ± 16.8 vs. 18.0 ± 2.8, *P* < 0.01, *n =* 6) (Figure 4C), possibly secondary to increased inflammation. Immunofluorescent staining confirmed increased α-smooth muscle actin and decreased adipocyte perilipin-1 expression in both EF and IF from macrophage COX-2^–/–^ mice (Figure 4, D and E, and Supplemental Figure 8A). Macrophage COX-2^–/–^ mice had increased plasma FFA levels 4 weeks after the HFD but decreased plasma FFA levels 12 weeks after the HFD, consistent with the increased fibrosis at this time point (Figure 4F).

In addition to fibrosis and unresolved inflammation, insufficient angiogenesis also contributes to the pathogenesis of dysfunctional adipose tissue in obesity (1). The mRNA levels of EF *Pecam1*/*Cd31*, a marker of vascular endothelial cells, were significantly lower in macrophage COX-2^–/–^ mice than WT mice after 12 weeks on the HFD (Figure 4G). CD31 immunofluorescent staining confirmed that macrophage COX-2^–/–^ mice had marked decreases in EF vascular density (Figure 4H). COX-2 has been reported to promote angiogenesis through induction of VEGF-A expression (17). Macrophage COX-2^–/–^ mice had markedly decreased EF *Vegfa* mRNA levels (Figure 4I), while the expression of the prolymphangiogenic factor *Vegfc* was not significantly altered (Supplemental Figure 8B). Immunofluorescent staining showed that VEGF-A was primarily expressed in ATMs in EF. Its expression was minimal in WT mice fed normal chow (Supplemental Figure 8C) but was evident in WT mice fed the HFD. In contrast, there was minimal VEGF-A expression in EF ATMs in macrophage COX-2^–/–^ mice on the HFD (Figure 4J).

### ATMs with COX-2^–/–^ deficiency decreased their ability to clear apoptotic adipocytes.

**Figure 5:**
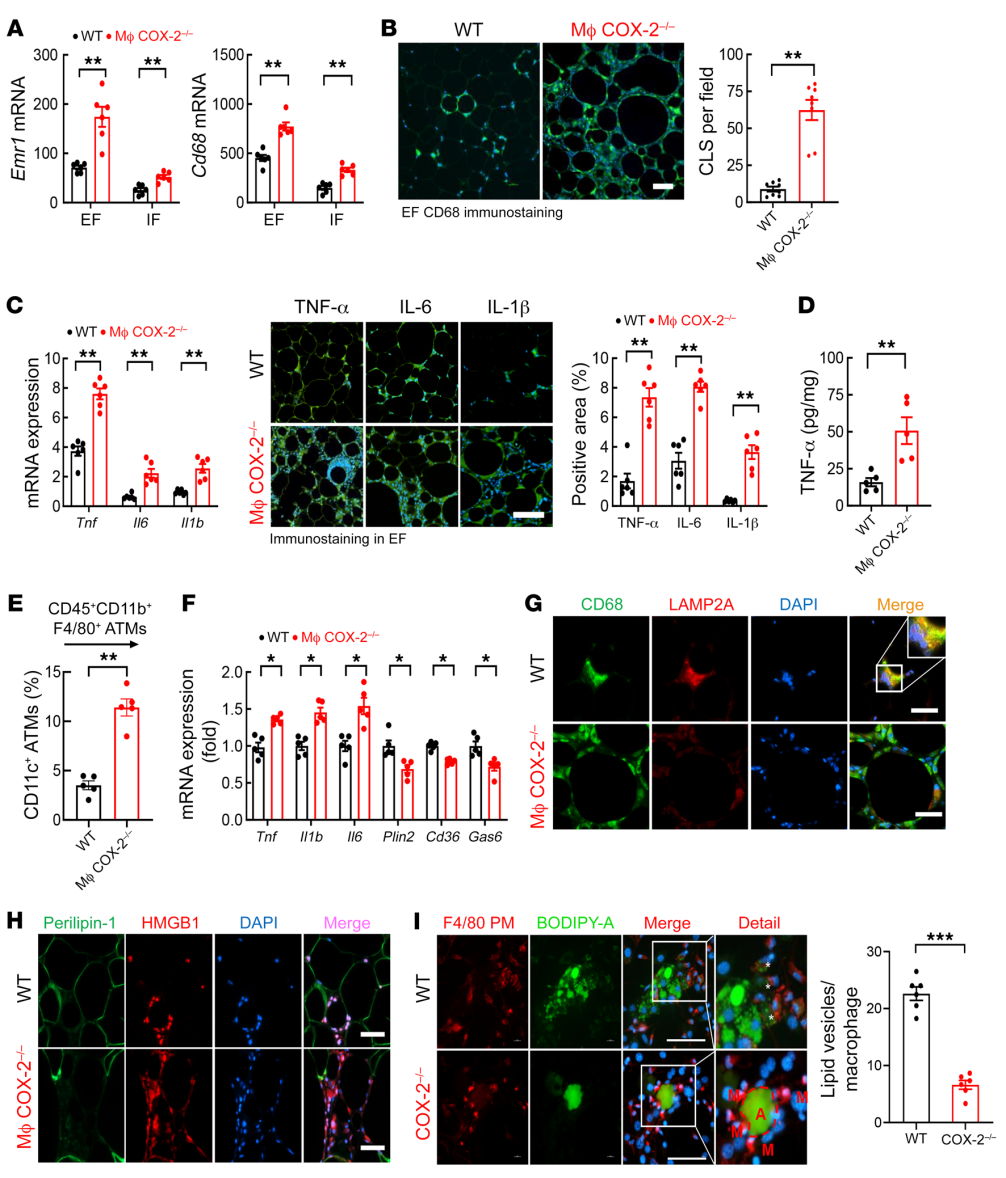
Myeloid COX-2^–/–^ mice had increased ATMs with impaired ability to phagocytose apoptotic adipocytes in DIO. WT and myeloid COX-2^–/–^ mice were on the HFD for 12 weeks. (**A**) Myeloid COX-2^–/–^ mice had increased *Emr1* and *Cd68* expression in EF and IF (*n =* 6). (**B**) Myeloid COX-2^–/–^ mice had increased CLSs in EF, as indicated by CD68 staining (*n =* 8). Scale bar: 100 μm. (**C**) Myeloid COX-2^–/–^ mice had increased proinflammatory mRNA and protein expression of TNF-α, IL-6, and IL-1β in EF (*n =* 5 and 6). Scale bar: 200 μm. (**D**) ELISA determined higher EF TNF-α expression in myeloid COX-2^–/–^ mice (*n =* 5). (**E**) Flow cytometry showed more proinflammatory ATMs (CD45^+^CD11b^+^F4/80^+^CD11c^+^) in myeloid COX-2^–/–^ mice (*n =* 5). (**F**) EF ATMs isolated from myeloid COX-2^–/–^ mice had increased proinflammatory cytokines (*Tnf*, *Il6*, and *Il1b*) but decreased expression of genes relating to lipid metabolism (*Plin2*, *Cd36*, and *Gas6*), indicating impaired ATM MMe polarization (*n =* 5). (**G**) Representative images showed that EF ATMs from myeloid COX-2^–/–^ mice had decreased expression of *LAMP2A*, a marker of MMe polarization. Scale bars: 50 μm. (**H**) HMGB1 was localized to nuclei of adipocytes and stromal cells in WT mouse EF but was detected in nuclei and cytosol in myeloid COX-2^–/–^ mice. Scale bars: 50 μm. (**I**) In vitro, the ability to phagocytose apoptotic adipocyte contents (green) was impaired in COX-2^–/–^ PMs (PMs, F4/80 staining, red) isolated from myeloid COX-2^–/–^ mice, as indicated by decreased lipid vesicles (asterisks) (*n =* 6). A, adipocytes; M, peritoneal macrophages. Scale bars: 50 μm. Data are mean ± SEM. *P < 0.05, **P < 0.01, ***P < 0.001, analyzed using 2-way ANOVA followed by Bonferroni’s post hoc test for **A** and 2-tailed Student’s *t* test for **B**–**F** and **I**. EF, epididymal fat; IF inguinal fat.

Macrophage COX-2^–/–^ mice had increased mRNA expression of macrophage markers *Emr1* and *Cd68* in both EF and IF (Figure 5A). In DIO, ATMs form CLSs to clear surrounding dead or dying adipocytes. COX-2 plays an important role in macrophage antiinflammatory polarization with an increased phagocytotic capacity (18–21). Macrophage COX-2^–/–^ mice had increased EF CLS numbers (Figure 5B) and mRNA and protein levels of proinflammatory cytokines, including TNF-α, IL-6, and IL-1β (Figure 5, C and D). Flow cytometry confirmed increased proinflammatory CD45^+^CD11b^+^F4/80^+^CD11c^+^ ATMs in macrophage COX-2^–/–^ mice on the HFD (Figure 5E). Although there were less inflammation and lower CLS numbers in IF, there were the same relative increases in macrophage COX-2^–/–^ mice (Supplemental Figure 9, A and B).

Compared with WT mice, ATMs isolated from macrophage COX-2^–/–^ mice had decreased expression of genes relating to lipid metabolism and phagocytosis, including *Plin2*, *Cd36*, and *Gas6*, and increased expression of proinflammatory cytokines, including *Tnf*, *Il6*, and *Il1b* (Figure 5F). In EF ATMs, the expression of LAMP2A, a marker of MMe polarization (13), was decreased in macrophage COX-2^–/–^ mice on the HFD (Figure 5G). Failure to clear apoptotic adipocytes can lead to secondary necrosis. High mobility group box 1 (HMGB1) released from the nucleus serves as a danger-associated molecular pattern signal. When apoptotic cells enter the phase of secondary necrosis, HMGB1 is released and can induce further inflammatory injury (22). HMGB1 was primarily localized in the nuclei in adipocytes and interstitial cells (primarily ATMs) in WT mice on the HFD for 12 weeks, but it could be detected in the nuclei and cytosol in macrophage COX-2^–/–^ mice (Figure 5H).

To evaluate phagocytotic ability in vitro, apoptotic adipocytes prestained with BODIPY (green) were cocultured with PMs prestained with F4/80 (red). PMs containing adipocyte-derived lipid could be observed in WT PMs, indicating effective phagocytosis, but lipid accumulation was markedly impaired in COX-2^–/–^ PMs (Figure 5I). COX-2 inhibition also impaired the ability of THP1 and RAW264.7 cells to efficiently phagocytose apoptotic adipocytes (Supplemental Figure 10).

### Myeloid COX-2 deficiency led to increased monocyte recruitment and ATM proliferation in adipose tissue in DIO.

**Figure 6:**
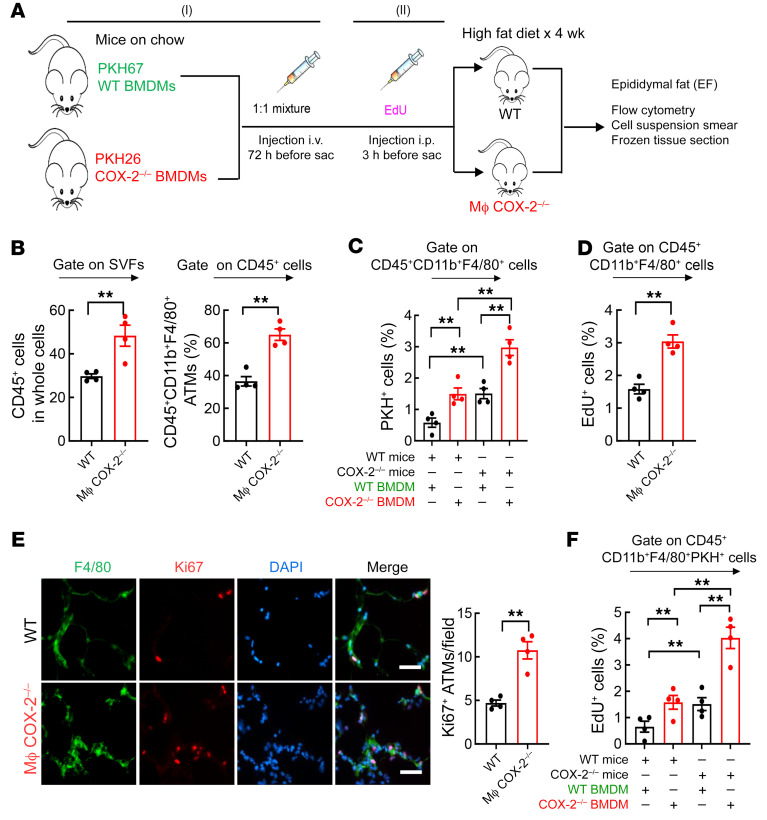
Increased monocyte recruitment and proliferation of ATMs contributed to more ATM accumulation in the HFD-treated myeloid COX-2^–/–^ mice. WT and myeloid COX-2^–/–^ mice were on the HFD for 4 weeks and EF was used for experiments. (**A**) Schematic of experimental protocol. (**B**) The percentage of CD45^+^ live cells and CD45^+^CD11b^+^F4/80^+^ ATMs in EF were markedly higher in myeloid COX-2^–/–^ mice than in WT mice (*n =* 4). (**C**) COX-2^–/–^ BMDMs had increased EF infiltration in both WT and myeloid COX-2^–/–^ recipients, and myeloid COX-2^–/–^ recipients had increased WT and COX-2^–/–^ monocyte recruitment (*n =* 4). (**D**) Myeloid COX-2^–/–^ mice had increased EF ATM proliferation rates. *n =* 4. (**E**) Myeloid COX-2^–/–^ mice had more Ki67-positive EF ATMs (*n =* 4). Scale bars: 50 μm. (**F**) Both infiltrating WT and COX-2^–/–^ BMDMs had greater EdU incorporation in myeloid COX-2^–/–^ than WT recipients (*n =* 4). Data are mean ± SEM. **P < 0.01, analyzed using 2-tailed Student’s *t* test for **B**, **D**, and **E**, and 2-way ANOVA followed by Bonferroni’s post hoc test for **C** and **F**. EF, epididymal fat.

To investigate the role of COX-2 in myeloid infiltration of EF, we labeled WT bone marrow–derived macrophages (BMDMs) with the monocyte tracking dye PKH67 (green) and COX-2^–/–^ BMDMs with PKH26 (red) and injected a mixture containing equal amounts of labeled WT and COX-2^–/–^ BMDMs into WT or macrophage COX-2^–/–^ recipients fed the HFD for 4 weeks (Figure 6A, part I). Flow cytometry confirmed increased EF CD45^+^CD11b^+^F4/80^+^ ATMs in macrophage COX-2^–/–^ mice at this time point (Supplemental Figure 11 and Figure 6B). In EF, the percentage of PKH26^+^ cells (COX-2^–/–^) was higher than that of PKH67-positive cells (WT) in WT and macrophage COX-2^–/–^ recipients, and the percentage of PKH67^+^ ATMs and PKH26^+^ ATMs was higher in macrophage COX-2^–/–^ recipients than in WT recipients (Figure 6C). More PKH26^+^ COX-2^–/–^ BMDMs were also observed in cell suspension smears from myeloid COX-2^–/–^ recipients (Supplemental Figure 12A). These results indicated that macrophage COX-2^–/–^ adipose tissue facilitated monocyte recruitment, and COX-2^–/–^ monocytes were more responsive to homing cues from the adipose tissue.

To investigate whether there were also differences in ATM proliferation, Click-iT Plus EdU Alexa Fluor 647 was i.p. injected 3 hours before euthanization to evaluate cells in the S phase of proliferation (Figure 6A, part II). EdU expression in EF ATMs was significantly higher in macrophage COX-2^–/–^ mice than WT mice (Figure 6D). Macrophage COX-2^–/–^ mice also had increased percentages of ATMs that were positive for Ki67, which was expressed at all stages of cell proliferation from G1 through mitosis (Figure 6E). A combination of strategies (I) and (II) indicated that infiltrating COX-2^–/–^ BMDMs had higher rates of proliferation, which was further increased in recipient macrophage COX-2^–/–^ mice (Figure 6F). An increase in proliferating PKH26 cells (EdU and PKH26-double positive) was confirmed by immunofluorescence in EF in macrophage COX-2^–/–^ mice (Supplemental Figure 12B).

### Mice with selective EP4 deletion in myeloid cells recapitulated the phenotype of mice with selective COX-2 deletion in myeloid cells in DIO.

**Figure 7:**
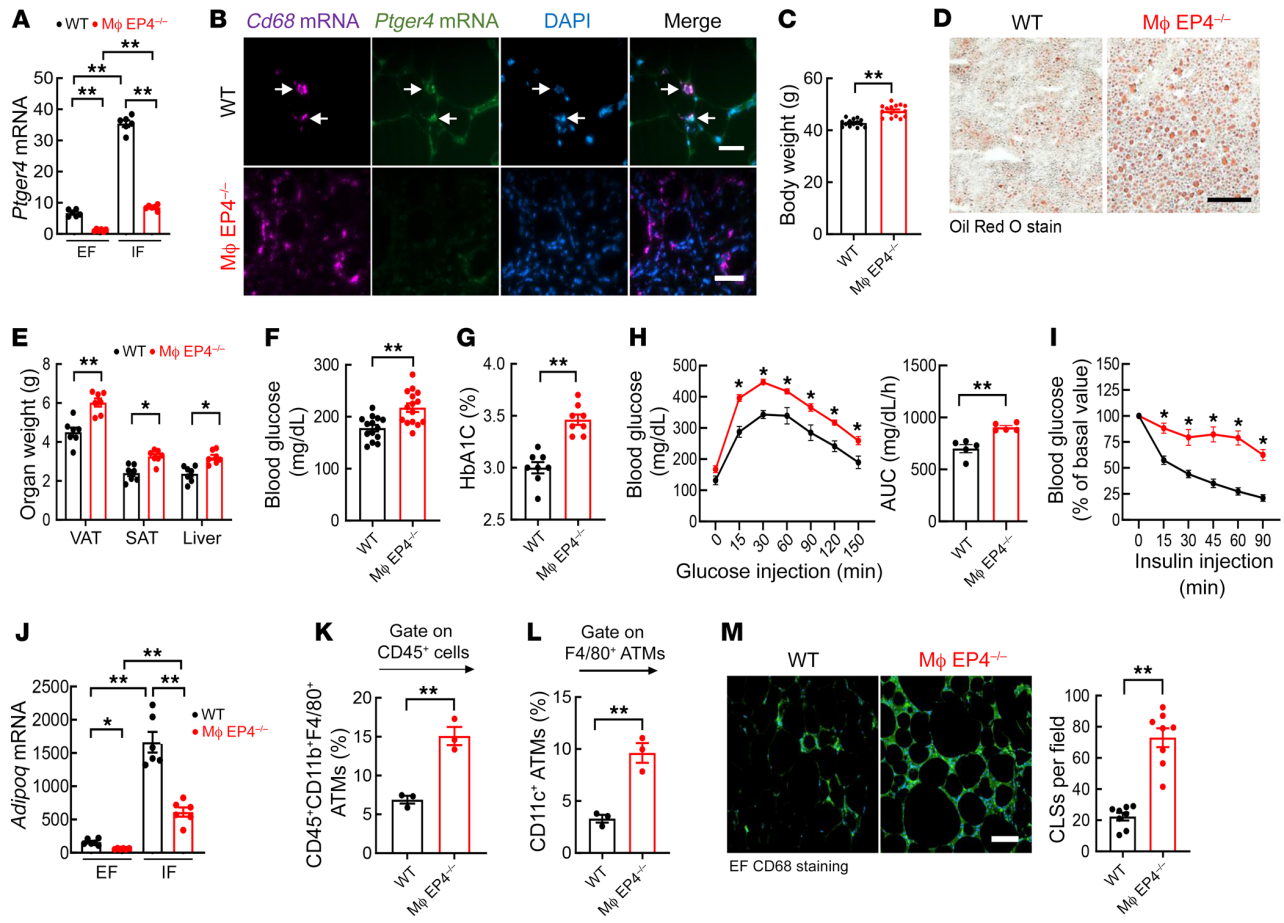
Mice with myeloid EP4 deletion recapitulated the phenotype of macrophage COX-2^–/–^ mice in DIO. WT (EP4^fl/fl^) and myeloid EP4^–/–^ (CD11b-Cre EP4^fl/fl^) mice were fed the HFD for 12 weeks. (**A**) *Ptger4* mRNA expression was more than 5 times lower in EF than IF in WT mice, and its expression was markedly decreased in both EF and IF in myeloid EP4^–/–^ mice (*n =* 6). (**B**) In situ hybridization indicated that *Ptger4* mRNA was colocalized with *Cd68* mRNA in ATMs in WT mice (arrows) but was not detectable in myeloid EP4^–/–^ mice. Scale bars: 50 μm. (**C**–**E**) Myeloid EP4^–/–^ mice had greater increases in body weight (*n =* 15) (**C**), liver steatosis (**D**), and SAT, VAT, and liver masses (*n =* 7) (**E**). Scale bar: 100 μm. (**F** and **G**) Myeloid EP4^–/–^ mice had greater increases in fasting blood glucose (*n =* 15) (**F**) and HbA1c (*n =* 8) (**G**). (**H** and **J**) Myeloid EP4^–/–^ mice had decreased glucose tolerance (*n =* 9) (**H**) and insulin sensitivity (*n =* 9) (**I**), and decreased *Adipoq* expression in both EF and IF (*n =* 6) (**J**). (**K**–**M**) Flow cytometry analysis showed more EF ATMs (*n =* 3) (**K**) with more proinflammatory phenotype (*n =* 3) (**L**) and higher number of crown-like structures (*n =* 8) (**M**) in myeloid EP4^–/–^ mice. Scale bar: 100 μm. Data are mean ± SEM. *P < 0.05, **P < 0.01, analyzed using 2-way ANOVA followed by Bonferroni’s post hoc test for **A** and **J**; 2-tailed Student’s *t* test for **C**, **E**–**G**, **K**–**M**; 2-way ANOVA followed by Tukey’s post hoc test for **I**; and 2-tailed Student’s *t* test and 2-way ANOVA followed by Tukey’s post hoc test for **H**. EF, epididymal fat; IF inguinal fat; subcutaneous and visceral adipose tissue (SAT and VAT).

Activation of the PGE2 receptor subtype EP4 can mediate macrophage polarization to inhibit proinflammatory cytokines/chemokines (18–20). We investigated the role of myeloid EP4 in the pathogenesis of DIO by generating CD11b-Cre EP4^fl/fl^ (macrophage EP4^–/–^) mice. Compared with WT (EP4^fl/fl^) mice, *Ptger4* (EP4) mRNA expression was markedly decreased in both EF and IF in macrophage EP4^–/–^ mice 12 weeks after initiation of the HFD (Figure 7A). Of note, *Ptger4* mRNA expression was markedly higher in IF than EF in both WT and macrophage EP4^–/–^ mice. Because reliable antibodies against EP4 receptors are not available, we utilized RNAscope to confirm colocalization of *Ptger4* mRNA with *Cd68* mRNA in CLSs in EF from WT mice and determined that *Ptger4* mRNA was not detectable in CLSs from macrophage EP4^–/–^ mice, although more *Cd68*-positive ATMs were present in CLSs (Figure 7B). Interestingly, EF *Ptger4* mRNA expression was also decreased in ATMs in macrophage COX-2^–/–^ mice on the HFD, suggesting a feed-forward regulation (Supplemental Figure 13).

There were no differences in body weights, fasting blood glucose, HbA1c, or glucose or insulin tolerance tests between WT and macrophage EP4^–/–^ mice after 20 weeks on the normal chow diet (Supplemental Figure 2). However, after 12 weeks of the HFD, macrophage EP4^–/–^ mice had greater body weight gains on the HFD than WT mice (Figure 7C); more severe liver steatosis (Figure 7D); increased SAT, VAT, and liver mass (Figure 7E); greater increases in fasting blood glucose (Figure 7F) and HbA1c levels (Figure 7G); abnormal glucose tolerance tests (Figure 7H) and insulin tolerance tests (Figure 7I); lower *Adipoq* mRNA levels (Figure 7J); an increased number of ATMs with a more proinflammatory CD45^+^CD11b^+^F4/80^+^CD11c^+^ phenotype (Figure 7, K and L, and Supplemental Figure 14A); and increased CLSs (Figure 7M). Similar to macrophage COX-2^–/–^ mice, macrophage EP4^–/–^ mice also demonstrated abnormal glucose tolerance and insulin tolerance after only 4 weeks of the HFD (Supplemental Figure 15).

**Figure 8:**
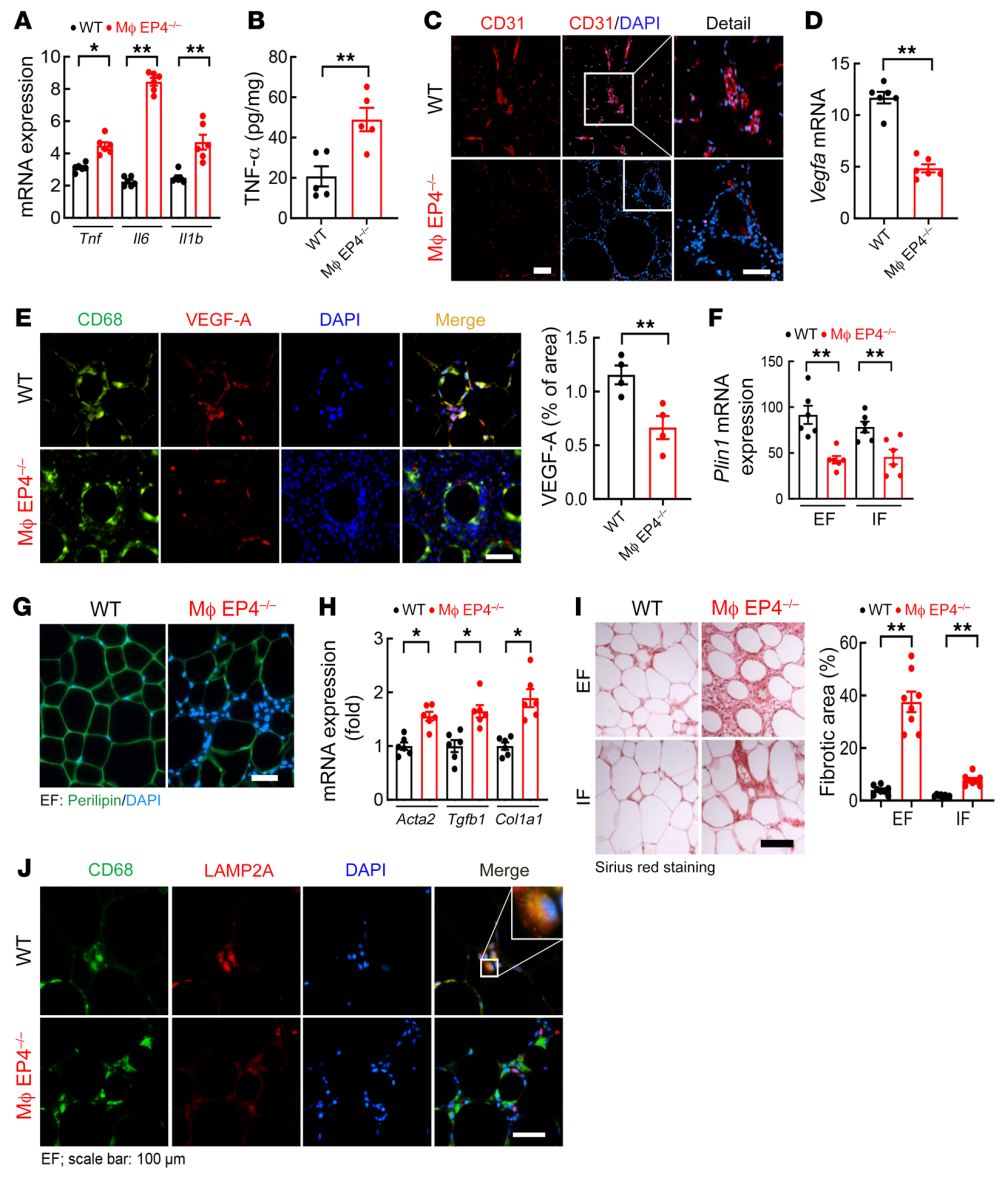
Mice with myeloid EP4 deletion had similar ATM dysfunction as myeloid COX-2^–/–^ mice. WT mice and myeloid EP4^–/–^ mice were fed with the HFD for 12 weeks. (**A** and **B**) Myeloid EP4^–/–^ mice had higher EF mRNA levels of proinflammatory cytokines (*Tnf*, *Il6*, and *Il1b*) (**A**) and higher EF TNF-α protein levels (**B**) (*n =* 5 and 6). (**C**) CD31 immunofluorescent staining showed less vascular density in myeloid EP4^–/–^ mice. Scale bars: 100 μm (left) and 50 μm (right). (**D** and **E**) Myeloid EP4^–/–^ mice had decreased EF *Vegfa* mRNA expression (**D**) and decreased EF ATM VEGF-A expression (**E**) (*n =* 4 and 6). Scale bar: 50 μm. (**F** and **G**) Myeloid EP4^–/–^ mice had decreased adipose tissue perilipin 1 (*Plin1*) mRNA (**F**) and protein (**G**) expression (*n =* 6). Scale bar: 100 μm. (**H**) Myeloid EP4^–/–^ mice had increased EF mRNA expression of *Acta2*, *Tgfb1*, and *Col1a1* (*n =* 6). (**I**) Myeloid EP4^–/–^ mice had significantly increased fibrosis in both EF and IF, with more than 4-fold higher level of fibrosis in EF compared with IF (*n =* 8). Scale bar: 100 μm. (**J**) Myeloid EP4^–/–^ mice had decreased EF ATM expression of *LAMP2A*, a marker of ATM MMe polarization. Scale bar: 100 μm. Data are mean ± SEM. *P < 0.05, **P < 0.01, by 2-tailed Student’s *t* test for **A**, **B**, **D**, **E**, and **H**, and 2-way ANOVA followed by Bonferroni’s post hoc test for **F** and **I**. EF, epididymal fat; IF inguinal fat.

The HFD-treated macrophage EP4^–/–^ mice also had increased adipose tissue proinflammatory cytokines (Figure 8, A and B, and Supplemental Figure 14B) and increased adipocyte size (Supplemental Figure 16), decreased vascular density (Figure 8C), decreased EF *Vegfa* mRNA expression (Figure 8D), and decreased VEGF-A protein expression in ATMs (Figure 8E). Macrophage EP4^–/–^ mice had decreased *Plin1* mRNA and protein expression (Figure 8, F and G) and increased adipose tissue fibrosis (Figure 8, H and I). Similar to myeloid COX-2 deletion, myeloid EP4 deletion also led to impaired ATM MMe polarization as indicated by decreased LAMP2A expression in epididymal ATMs (Figure 8J). In addition, EP4^–/–^ PMs isolated from myeloid EP4^–/–^ mice had impaired ability to phagocytose apoptotic adipocytes (Supplemental Figure 17).

### Activation of EP4 receptors ameliorated insulin resistance and metabolic syndrome seen in myeloid COX-2^–/–^ mice but not in myeloid EP4^–/–^ mice.

**Figure 9:**
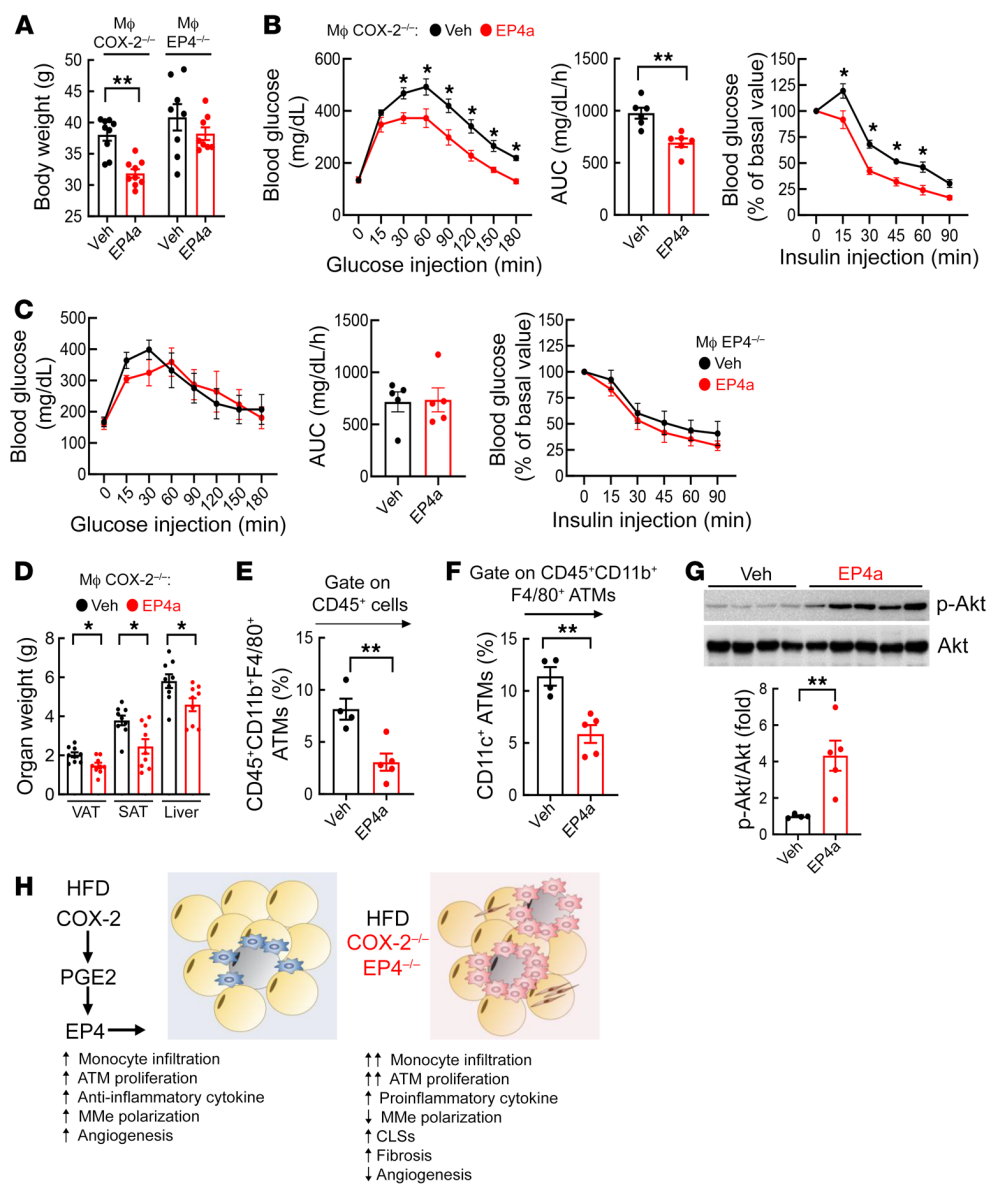
An EP4 agonist ameliorated HFD-induced metabolic syndrome in myeloid COX-2^–/–^ mice but not in myeloid EP4^–/–^ mice. Myeloid COX-2^–/–^ mice and myeloid EP4^–/–^ mice were treated with the HFD for 10 weeks with or without the selective EP4 agonist (EP4a). (**A**) The EP4a attenuated the HFD-induced gain of body weight in myeloid COX-2^–/–^ mice but not in myeloid EP4^–/–^ mice (*n =* 8–9). (**B** and **C**) The EP4a improved glucose and insulin tolerance in myeloid COX-2^–/–^ mice (**B**) but not in myeloid EP4^–/–^ mice (**C**) (*n =* 8). (**D**) The EP4a decreased SAT, VAT, and liver mass in the HFD-treated myeloid COX-2^–/–^ mice (*n =* 9). (**E** and **F**) Flow cytometry demonstrated that the EP4a decreased EF ATM number (**E**) and proinflammatory polarization (CD45^+^CD11b^+^F4/80^+^CD1c^+^) (**F**) in myeloid COX-2^–/–^ mice (*n =* 4 and 5). (**G**) Immunoblotting showed increased EF insulin-stimulated p-AKT in the EP4a-treated myeloid COX-2^–/–^ mice (*n =* 4 and 5). (**H**) Graphical summary of the current studies. Data are mean ± SEM. *P < 0.05, **P < 0.01, analyzed using 2-tailed Student’s *t* test for **A** and **D**–**G**, and 2-tailed Student’s *t* test and 2-way ANOVA followed by Tukey’s post hoc test for **B** and **C**. EF, epididymal fat; subcutaneous and visceral adipose tissue (SAT and VAT).

WT mice, macrophage COX-2^–/–^ mice, and macrophage EP4^–/–^ mice were fed the HFD with or without the EP4 agonist, ONO-4819, which was given via mini-pump at a dose of 75 μg/kg/day throughout the 10-week experimental period. Although the EP4 agonist did not significantly affect metabolic readouts in WT mice (Supplemental Figure 18), it led to lower body weights (Figure 9A); improved glucose tolerance tests and insulin tolerance tests (Figure 9B); and decreased SAT, VAT, and liver mass in macrophage COX-2^–/–^ mice (Figure 9D). In contrast, the EP4 agonist had no effect on body weight or glucose tolerance tests and insulin tolerance tests in macrophage EP4^–/–^ mice (Figure 9, A and C). Flow cytometry determined that both total ATMs and proinflammatory CD45^+^CD11b^+^F4/80^+^CD11c^+^ ATMs were decreased in EP4 agonist–treated macrophage COX-2^–/–^ mice (Figure 9, E and F). Increased EF insulin-activated p-Akt levels confirmed increased insulin sensitivity in EP4 agonist–treated macrophage COX-2^–/–^ mice (Figure 9G).

## Discussion

The current studies demonstrated that in response to an HFD, ATMs increased COX-2 expression. Palmitic acid, the major FFA released by adipocytes, stimulated COX-2 expression in mouse PMs and in both human and mouse macrophage-like cell lines. Selective deletion of COX-2 expression in ATMs increased EF and IF deposition, with increased peripheral insulin resistance. COX-2 deletion in ATMs induced a more proinflammatory phenotype and inhibited the ability to phagocytose lipid contents from apoptotic adipocytes. Selective myeloid COX-2 deficiency led to increased monocyte recruitment to adipose tissue and increased proliferation of ATMs in DIO. Macrophage expression of the PGE2 EP4 receptor was inhibited by COX-2 deletion, and selective myeloid deletion of EP4 produced a similar phenotype to that seen with myeloid deletion of COX-2, with a more proinflammatory ATM phenotype, increased fat deposition, insulin resistance, and inhibition of adipocyte phagocytosis (Figure 9H). EP4 expression was relatively greater in superficial inguinal adipose tissue than in epididymal adipose tissue, consistent with increased metabolic abnormalities with visceral adiposity (23). These results indicate a role for a COX-2/PGE2/EP4 axis to modulate the phenotype of ATMs to lessen the complications of obesity.

In the current studies, the lack of efficacy of the EP4 agonist in mice with myeloid EP4 deletion indicated that the predominant effect of the EP4 agonist was its ability to ameliorate the proinflammatory ATM phenotype. In this regard, previous studies by us and others have indicated that deletion of macrophage-dependent COX-2 expression can mediate macrophage polarization to a proinflammatory M1 phenotype in resident kidney tissue macrophages (18, 24), and EP4 activation in macrophages inhibits macrophage cytokine and chemokine release (8, 19, 20, 25–27).

Prior studies by the Scherer group have identified 3 factors leading to dysfunctional adipose tissue: persistent inflammation, inappropriate extracellular matrix deposition, and abnormalities in angiogenesis (1, 2). In the current studies, selective myeloid deletion of either COX-2 or EP4 induced all 3 elements in adipose tissue: ATMs from these mice had increased expression of proinflammatory cytokines, increased expression of TGF-β and fibrosis, and impaired expression of VEGF-A, indicating an important role for the COX-2/EP4 axis to serve as a brake on development of adipose tissue dysfunction in response to high-fat feeding. Although COX-2 expression has been implicated in regulation of macrophage-derived VEGF-A in tumor angiogenesis (17), these studies demonstrated an important role for COX-2 regulation of macrophage-derived VEGF-A in angiogenesis in adipose tissue.

One of the most important functions of ATMs in adipose tissue is to remove apoptotic adipocytes (3). With obesity, metabolically active MMe ATMs surround apoptotic adipocytes in the CLSs. Macrophages are only one-sixth or less the size of adipocytes, so traditional methods of efferocytosis of apoptotic adipocytes are not possible. Previous studies have demonstrated that ATMs attach to the apoptotic adipocytes and inject lysosomal lipases that release FFAs that are then phagocytosed by the macrophages (13, 28). In the current studies, we found that although selective deletion of myeloid COX-2 or EP4 led to increased formation of CLSs, the ATMs had decreased expression of multiple components of macrophage phagocytotic machinery and limited ability to phagocytose lipid contents from apoptotic adipocytes.

Increased adipose lipolysis and increased plasma FFA and glycerol due to adipose inflammation may contribute to the insulin insensitivity and abnormal glucose disposal observed in the macrophage COX-2^–/–^ mice. Previous studies have clearly demonstrated that adipose tissue inflammation increases lipolysis (29). With deletion of COX-2 in ATMs, we also found increased FFAs and glycerol after 4 weeks on the HFD. The relative decrease seen at 12 weeks was presumably due to the increased adipose tissue fibrosis and a decreased number of viable adipocytes. We also cannot discount the possibility that increased inflammation in other tissues in the macrophage COX-2^–/–^ mice may also play a role in insulin resistance. In addition, a recent study has also indicated a role for resident islet macrophages to affect β cell function in high fat–induced obesity (30).

Prior studies investigating the potential role of COX-2 in adipocytes have provided contradictory results. In models of high fat–induced obesity, adipocyte COX-2 expression was reported to increase (31), and administration of selective COX-2 inhibitors or global COX-2 gene deletion reduced adipose tissue mass and inflammation and skeletal muscle insulin resistance (31–34). Adipose MIF (migration inhibitory factor) has been reported to be mediated by COX-2 activity and to mediate adipose macrophage polarization to an M1 phenotype (35). Selective deletion of adipose phospholipase A2, the mediator of arachidonic release, prevented development of high fat–induced obesity (36). In vitro studies suggested that adipocyte-derived PGE2 may mediate macrophage recruitment (37), and mice with mPGES-1 deletion, which mediates PGE2 synthesis, had decreased weight gain and adipose inflammation when fed an HFD (38). In contrast, a recent study investigating adipocyte-specific overexpression of COX-2 reported a small selective decrease in dietary high fat–induced inguinal adipose tissue mass, with no change in glucose tolerance and marginal improvement in insulin sensitivity (39). In our studies, we also failed to detect any significant effect on blood glucose or glucose tolerance as well as any significant difference in total body weight for the HFD in mice with selective adipocyte COX-2 deletion. Therefore, although it remains unclear whether adipocyte-derived COX-2 has any involvement in mediating adiposity and inflammation, the current study clearly demonstrated that deletion of macrophage COX-2 will predispose to adipose inflammation due to a more proinflammatory ATM phenotype.

There is similar uncertainty in the literature about the effects of EP4. It has been reported that chronic administration of an EP4-selective agonist inhibited insulin resistance and adipose inflammation in a model of obesity-induced diabetes (*db/db* mice; ref. 40). A recent study reported that global EP4 deletion led to increased body weight when mice were fed a chow diet or an HFD but had no effect on ATM density and polarization. When mice were fed an HFD, either global or adipocyte-selective EP4 deletion stimulated adipose lipolysis and improved insulin sensitivity. The authors determined that adipocyte EP4 was primarily activated by COX-1–derived PGE2 (41). In contrast, the present study utilized mice with selective EP4 deletion in myeloid cells, and the differences in metabolic readouts were only observed when they were fed the HFD. Therefore, similar to COX-2, there appear to be phenotypic differences in effects seen with EP4 deletion depending on the cell type.

It should be emphasized that the current studies were limited to investigation in mice, and further studies will be required to determine whether there is a similar role for a COX-2/PGE2/EP4 axis in human ATMs. Previous studies have suggested that unlike mice, in which obesity induces a phenotypic switch of macrophages to a proinflammatory phenotype, in obese humans, ATMs may represent a mixed inflammatory phenotype characterized by the presence of markers classically associated with both an M1 and M2 phenotype (42). This mixed M1/M2 phenotype or MMe phenotype is metabolically active, increased lysosomal activity, and is associated with increased formation of CLSs and insulin resistance in obese humans (13, 43).

It is also noteworthy that in addition to the increased adiposity and insulin resistance, macrophage COX-2^–/–^ mice were noted to have increased food intake. Previous studies have indicated that centrally administered PGE2 inhibits food intake (44). High-fat feeding leads to development of a proinflammatory microglial phenotype in the mediobasal hypothalamus, a key regulatory site of food intake and satiety, which disrupts the normal physiological pattern of food intake as well as energy expenditure (45). COX-1 has been shown to be activated in microglia in brain inflammation and to mediate prostanoid release that further promotes inflammation. In contrast, upregulated microglial COX-2 expression promotes production of lipid mediators that promote resolution of inflammation (46). To obviate the potential effect of differences in weight gain on metabolic abnormalities, we pair-fed WT and macrophage COX-2^–/–^ mice the HFD for 4 weeks. Both groups had similar body weight gains, but COX-2^–/–^ mice still exhibited higher fasting blood glucose and greater abnormality of glucose tolerance tests and insulin tolerance tests.

In summary, these studies report a role for expression of COX-2 and its metabolite, PGE2, to mitigate adipose responses to an HFD. Selective deletion of ATM COX-2 expression or the PGE2 receptor subtype EP4 exacerbated a proinflammatory ATM phenotype and the metabolic abnormalities associated with obesity. Administration of an EP4 agonist improved the metabolic abnormalities by selectively correcting the metabolically activated ATM phenotype. Therefore, an ATM COX-2/PGE2/EP4 axis plays a protective role in response to an HFD.

## Methods

### Animals.

EP4^fl/fl^ mice were generated in Matthew Breyer’s laboratory (Vanderbilt University School of Medicine, Nashville, Tennessee, USA) (47), COX-2^fl/fl^ mice in Garret A. Fitzgerald’s laboratory (University of Pennsylvania, Philadelphia, Pennsylvania, USA) (48), CD11b-Cre mice with transgene integration in the Y chromosome in Jean Vacher’s laboratory (Institut de recherches cliniques de Montreal, Montreal, Quebec, Canada) (49), and all these mice were backcrossed onto the FVB background for 12 generations. Myeloid COX-2 deletion (CD11b-Cre COX-2^fl/fl^, macrophage COX-2^–/–^) and EP4 deletion (CD11b-Cre EP4^fl/fl^, macrophage EP4^–/–^) mice and corresponding WT mice (WT, COX-2^fl/fl^, and EP4^fl/fl^, respectively) on the FVB background were used for experiments. Adipoq-Cre mice were purchased from The Jackson Laboratory (stock 028020, C57BL/6J) and crossed with C57BL/6J COX-2^fl/fl^ mice to generate mice with selective COX-2 deletion in adipocytes (Adipoq-Cre COX-2^fl/fl^, adipocyte COX-2^–/–^). We used 2 controls for studies. For most of the studies, we used either COX-2^fl/fl^ or EP4^fl/fl^ as controls. In addition, we generated CD11b-Cre littermate mice by crossing female COX-2^fl/+^ with CD11b-Cre COX-2^fl/+^ to generate CD11b-Cre littermate controls and CD11b-Cre COX-2^fl/fl^ mice. CD11b-Cre mice and COX-2^fl/fl^ mice had similar responses to an HFD. Male 8- to 10-week-old mice were used for all experiments, and genotypes were confirmed with PCR before and after experiments. The primers used in this study included the primers for *Ptgs2* (COX-2) floxed mice: 5′-TGAGGCAGAAAGAGGTCCAGCCTT-3′ and 5′-ACCAATACTAGCTCAATAAGTGAC-3′; *Ptgs2* deletion allele. 5′-TGAGGCAGAAAGAGGTCCAGCCTT-3′ and 5′-TTTGCCACTGCTTGTACAGCAATT-3′. The primers for *Ptger4* (EP4) floxed mice were 5′-GTTAGATGGGGGGAGGGGACAACT-3′ and 5′-TCTGTGAAGCGAGTCCTTAGGCT-3′; the primers for *CD11b*-Cre mice were 5′-AATGCTTCTGTCCGTTTGC-3′ and 5′-CGGCAACACCATTTTTTCTG-3′; and the primers for *Adipoq*-Cre mice were 5′-ACGGACAGAAGCATTTTCCA-3′ and 5′-GGATGTGCCATGTGAGTCTG-3′. The HFD (F3282) was from Bio-Serv and the medium-fat diet (PicoLab 5LJ5 diet) was from LabDiet. For pair-feeding experiments, we housed 2 mice with similar body weights in each cage according to a protocol approved by Vanderbilt IACUC. We measured food intake as g/100 g body weight from WT mice and gave that amount to myeloid COX-2^–/–^ mice. Circadian rhythm was similar between WT and myeloid COX-2^–/–^ mice on chow food or HFD, with the majority of food intake during the night. We added food to the pair-feeding myeloid COX-2 mice at 8 pm, so the mice ate most of their food at night.

### Antibodies and reagents.

Rabbit anti–murine COX-2 (catalog 160106) was from Cayman Chemical; rat anti–mouse F4/80 (catalog ab6640), rabbit anti–VEGF-A (catalog ab52917), anti–IL-1β (catalog ab9722), and anti-Ki67 (catalog ab16667) were from Abcam; rat anti–mouse CD68 (catalog MCA1957B) was from Bio-Rad; rabbit anti-LAMP2A (catalog L0668), mouse anti–β-actin (catalog A1978), and anti–α-smooth muscle actin were from Sigma-Aldrich; mouse anti–IL-6 (catalog sc-32296) and anti–TNF-α (catalog sc-133192) were from Santa Cruz Biotechnology; rabbit anti–p-AKT (Ser473) (catalog 4046, clone D9E), anti-AKT (catalog 4685), anti–perilipin-1 (catalog 9449, clone D1D8), and HMGB1 (catalog 6893S) were from CST; and rabbit anti-CD31/PECAM-1 was from Novus Biologicals (catalog NB100-2284). The HFD was from Bio-Serv (36% fat accounting for 60% of calories, F3282); water-soluble dexamethasone (catalog D2951) and palmitic acid (catalog 506345) were from MilliporeSigma. For the in vitro study, 5 mM palmitate conjugated to 2 mM FFA-free BSA in sterile water (pH 7.4) was used. ONO-4819 (Rivenprost, LS-H93, LSbio) was given at 75 μg/kg/day via Alzet model mini-pump.

### Isolation of BMDMs, PMs, and fat tissue myeloid cells.

The mice were anesthetized with isoflurane and euthanized by cervical dislocation. Femurs, tibias, and humeri were dissected, and the shafts were flushed using a syringe and a 26-gauge needle with RPMI 1640 supplemented with 100 U/mL penicillin, 100 μg/mL streptomycin, 10 U/mL heparin, and 0.2% FBS. The cell suspension was passed through a 40-μm strainer and centrifuged, and the pellets were resuspended in 3 mL red blood cell lysis buffer and incubated for 3 minutes at room temperature. After centrifugation, the pellets were resuspended with 10 mL Dulbecco’s PBS containing 0.5% FBS, followed by monocyte isolation using a monocyte isolation kit for mice (Miltenyi Biotec, 130-100-629) (50). Adipose tissue myeloid cells were enriched using mouse CD11b microbeads and MACS columns (Miltenyi Biotec) following the manufacturer’s protocol. For isolation of PMs, mice were i.p. injected with 3 mL of sterile thioglycolate medium (3% w/v of an autoclaved stock prepared from dehydrated thioglycolate medium and sterile saline water) (Sigma-Aldrich). Three days later, peritoneal fluid was harvested, and pellets were resuspended in RPMI 1640 supplemented with 100 U/mL penicillin, 100 μg/mL streptomycin, 10 U/mL heparin, and 10% FBS and seeded in a 10-cm dish for 3 hours. After washing 3 times with culture medium, cells were used for study.

### Hyperinsulinemic-euglycemic clamp.

Mice were anesthetized under 2% (v/v) isoflurane and the left common carotid artery and the right jugular vein were catheterized for sampling and infusions, respectively, as previously described (51). On the day of the experiment, food was removed at 8 am. After 3.5 hours fasting, a primed (2 minutes, 0.5 μCi/min) followed by continuous infusion (0.05 μCi/min) of [3-^3^H] glucose was administered to measure whole-body glucose turnover. After 5 hours of fasting, mice received a continuous insulin infusion (4 mU/kg/min), and blood glucose was maintained at basal levels by a variable infusion of a 50% (w/v) glucose solution. Arterial blood samples were collected during steady-state conditions and at 80, 90, 100, 110, and 120 minutes for determination of the rate of whole-body glucose disappearance (Rd) and the rate of endogenous glucose appearance (Ra), as described above. At 120 minutes, a 13 μCi bolus of [^14^C]-2-deoxy-D-glucose (2-DG) was injected into the jugular vein, and arterial blood was sampled at 122, 135, 145, and 155 minutes. Mice were administered pentobarbital anesthesia, and tissues were extracted and frozen for subsequent gene expression and glucose uptake determinations.

### Flow cytometry.

EF tissue cell suspension was prepared using DNase I (56 U/mL, Bio-Rad, 7326828) and collagenase D (4 mg/mL, Roche, 11088882001), and then suspended in 100 μL of PBS containing 1% BSA and incubated with 0.5 μL Fc block (BD Pharmingen, purified rat anti-mouse CD16/CD32). Anti-CD45 (30-F11, 103149), anti-CD11b (M1/70, 101226), and anti-F4/80 (BM8, 123129) were used to identify ATMs, and anti-CD11c (N418, 117317) was used to label proinflammatory ATMs. All these reagents were from BioLegend. Appropriate isotype controls were included for each sample. A total of 100,000 cells were acquired by scanning using NovoCyte Quanteon Flow Cytometer Systems. Cell debris and dead cells were excluded from the analysis based on scatter signals and use of Zombie Violet Fixable Viability kit (423114, BioLegend). For evaluation of monocyte infiltration into adipose tissue, 100 μL of solution containing 1 × 10^6^ PKH67-labeled WT BMDMs (green, PKH67GL, Sigma-Aldrich) and 1 × 10^6^ PKH26-labeled COX-2^–/–^ BMDMs (from myeloid COX-2^–/–^ mice) (red, PKH26GL, Sigma-Aldrich) were injected retro-orbitally to either WT or myeloid COX-2^–/–^ mice on the HFD for 4 weeks, 72 hours before euthanization. Cell proliferation in S phase was evaluated using Click-iT Plus EdU Alexa Fluor 647 Flow Cytometry Assay kit (Invitrogen, C10634), which was administrated via i.p. injection 3 hours before euthanization according to the manufacturer’s protocol.

### RNAscope.

RNA in situ hybridization (RNAscope, ACD Bio-Techne) was performed on FFPE sections using the RNAScope Multiplex Fluorescent Reagent v2 kit RED according to the manufacturer’s instructions. Probes against mouse *Cd68* (Mm-Cd68-C2, ACD 316611-C2) and mouse *Ptger4* (EP4, Mm-Ptger4, ACD 441461) were used.

### Phagocytic activity assay.

3T3-L1 adipocytes (ATCC CL-173) were differentiated with IBMX-DEX-INS for 10 days and then induced to form apoptotic bait cells by staurosporine apoptosis inducer (1:1000) and prelabeled with BODIPY (1:2500, BODIPY 493/503, Invitrogen) to stain neutral lipids. Effector cells, including PMs, mouse macrophage-like RAW264.7 cells (ATCC TIB-71), and human macrophage-like THP1 cells (ATCC TIB-202) were prelabeled with F4/80 (1:50 staining) and plated on 24-well plates. Macrophages were layered on the top of adipocytes in a ratio of 4:1 and were cocultured for 24 hours at 37°C in a 5% CO_2_ atmosphere, and images were obtained with a Nikon TE300 fluorescence microscope and spot-cam digital camera (Diagnostic Instruments).

### Glucose tolerance test and insulin tolerance test.

For the glucose tolerance test, mice fasted overnight (16 hours) were i.p. injected with glucose at a dose of 2 mg/g body weight and blood glucose was measured 0, 15, 30, 90, 120, 150, and 180 minutes after glucose injection (52). For the insulin tolerance test, insulin was given at a dose of 3 μg/kg body weight to mice fasting from 8 am to 2 pm, and blood glucose was monitored 0, 15, 30, 45, 60, 75, and 90 minutes after insulin administration (52).

### Quantitative PCR.

Total RNA from tissues and cells was isolated using TRIzol reagent (Invitrogen). SuperScript IV First-Strand Synthesis System kit (Invitrogen) was used to synthesize cDNA from equal amounts of total RNA from each sample. Quantitative real-time PCR was performed using TaqMan real-time PCR (7900HT, Applied Biosystems). The Master Mix and all gene probes were also purchased from Applied Biosystems. The probes used in the experiments included the following mouse primers: *Gapdh* (Mm99999915), *Ptgs2* (Mm00478374), *Ptges* (mm00452105), *Ptger4* (Mm00436053), *Emr1* (Mm00802529), *Cd68* (Mm03047343), *Tnf* (Mm99999068), *Il1a* (Mm00439621), *Il1b* (Mm00434228), *Il6* (Mm00446190), *Ccl2* (Mm00441242), *Col1a1* (Mm00801666), *Col4a1* (Mm01210125), *Acta2* (Mm01546133), *Tgfb1* (Mm00441726), *Gas6* (Mm00490378), *Cd31* (Mm01242584), *Adipoq* (Mm00456425), *Cd36* (Mm00432403), *Plin1* (perilipin 1, Mm00558672), *Plin2* (perilipin 2, Mm00475794), *Vegfa* (Mm01281449), and *Vegfc* (Mm00437313). Human primers included *GAPDH* (Hs99999905) and *PTGS2* (Hs00153133). Real-time PCR data were analyzed using the 2^–ΔΔCT^ method to determine the fold difference in expression.

### Quantitative immunofluorescence staining.

Animals were anesthetized with Nembutal (70 mg/kg, i.p.) and given heparin (1000 U/kg, i.p.) to minimize coagulation. After perfusion with cold PBS through a transcardial aortic cannula, tissues were removed for immunoblotting, flow cytometry, quantitative PCR, immunofluorescent staining, isolation of renal myeloid cells, or histology with immersion in fixative containing 3.7% formaldehyde, 10 mM sodium *m*-periodate, 40 mM phosphate buffer, and 1% acetic acid. To determine insulin sensitivity in different organs with p-Akt immunoblotting and immunofluorescent staining, insulin at a dose of 1.5 U/kg was given i.p. 5 minutes before euthanization. The fixed tissue was dehydrated through a graded series of ethanol, embedded in paraffin, sectioned (5 μm), and mounted on glass slides. The deparaffinized sections underwent antigen retrieval with citrate buffer by microwave heat for 10 minutes, and then were blocked with 10% normal donkey serum for 1 hour at room temperature. For double immunofluorescence staining, the sections were incubated in 2 rounds of staining: CD68 (1:100 dilution) versus COX-2 (1:100 dilution) or VEGF-A (1:50 dilution) or LAMP2A (1:50 dilution); perilipin-1 (1:100 dilution) versus HMGB1 (1:100 dilution) overnight at 4°C, followed by either anti–rabbit IgG–HRP or anti–mouse IgG–HRP incubation at room temperature for 1 hour. Each round was followed by tyramide signal amplification with Alexa Flour 488 tyramide or Alexa Flour 555 tyramide (Tyramide SuperBoost kit with Alexa Fluor tyramides, Invitrogen) according to the manufacturer’s protocols. DAPI was used as a nuclear stain. Of note, 5-μm cryosections from fresh tissue embedded in Tissue-Tek OCT were used for PKH26, PKH67, and EdU evaluation as well as F4/80 and Ki67 double immunofluorescent staining in fat tissues. Sections were viewed and imaged with a Nikon TE300 fluorescence microscope and spot-cam digital camera (Diagnostic Instruments), followed by quantification using ImageJ (NIH) in more than 10 fields per slide and expressed as arbitrary units or percentage per field by 2 independent investigators.

### Immunoblotting analysis.

Skeletal muscle, liver, PMs, RAW 264.7, and THP1 cells were homogenized using lysis buffer containing 10 mM Tris-HCl (pH 7.4), 50 mM NaCl, 2 mM EGTA, 2 mM EDTA, 0.5% Nonidet P-40, 0.1% SDS, 100 μM Na_3_VO_4_, 100 mM NaF, 0.5% sodium deoxycholate, 10 mM sodium pyrophosphate, 1 mM PMSF, 10 μg/mL aprotinin, and 10 μg/mL leupeptin and centrifuged at 15,000*g* for 20 minutes at 4°C (53). Adipose tissue protein was extracted with Minute Total Protein Extraction kit (Invent biotechnologies). The BCA protein assay kit (Thermo Fisher Scientific) was used to measure the protein concentration. Immunoblotting was performed as previously described (53) and quantitated with ImageJ.

### Evaluation of adipocyte size and lipid accumulation in macrophages.

Adipocyte diameter was measured in Picrosirius red–stained slides with ImageJ. Sixty-five adipocytes were analyzed for each of 6 mice from each group. Gaussian distribution was added to illustrate the normal distribution of the data set. Lipid vesicles from 20 to 25 macrophages were quantitated and the mean was used for each sample.

### Measurement of plasma insulin, FFA, glycerol, and tissue TNF-α.

Ultra Sensitive Mouse Insulin ELISA kit (900800, Crystal Chem) was used to measure plasma insulin concentration according to the manufacturer’s protocol. FFA quantitation kit (MAK044) and glycerol assay kit (MAK117) from Sigma-Aldrich were used to measure plasma FFA and glycerol according to the manufacturer’s protocol. Mouse fat tissue TNF-α levels were evaluated using mouse TNF-α DuoSet ELISA kit (DY410, R&D Systems).

### HbA1c evaluation.

HbA1c levels were measured using a DCA Vantage Analyzer (Siemens).

### Oil Red O and Picrosirius red staining.

Frozen liver tissues were embedded in Tissue-Tek OCT and 10-μm thick cryosections were used for Oil Red O staining to detect neutral lipid accumulation using an Oil Red O Stain Kit (Abcam, ab150678). Fat tissue fibrosis was determined using a Picrosirius red stain kit (Direct Red 80, 365548, Sigma-Aldrich).

### Statistics.

Statistical analyses were performed with GraphPad Prism 9. Data are presented as the mean ± SEM. Data were analyzed using a 2-tailed Student’s *t* test, 1-way ANOVA, or 2-way ANOVA followed by Tukey’s or Bonferroni’s post hoc tests. A *P* value less than 0.05 was considered significant. For each set of data, at least 4 animals were examined for each condition. Collection, analysis, and interpretation of data were conducted by at least 2 independent investigators, who were blinded to the study.

### Study approval.

All animal experiments were performed in accordance with the guidelines of the IACUC of Vanderbilt University.

## Author contributions

MZZ and RCH conceived the study. YP, SC, JT, DHW, YW, AN, XF, MJ, YZ, and SW performed the experiments. YP, SC, MZZ, and RCH prepared the figures. YP, JPAO, AST, DHW, MZZ, and RCH wrote and edited the manuscript.

## Supplementary Material

Supplemental data

## Version 1 05/02/2022

Electronic publication

## References

[1] Crewe C (2017). The ominous triad of adipose tissue dysfunction: inflammation, fibrosis, and impaired angiogenesis. J Clin Invest.

[2] Sun K (2013). Fibrosis and adipose tissue dysfunction. Cell Metab.

[3] Boutens L, Stienstra R (2016). Adipose tissue macrophages: going off track during obesity. Diabetologia.

[4] Hill AA (2014). A decade of progress in adipose tissue macrophage biology. Immunol Rev.

[5] Antony A (2021). Deficiency of Stat1 in CD11c^+^ cells alters adipose tissue inflammation and improves metabolic dysfunctions in mice fed a high-fat diet. Diabetes.

[6] Zhang MZ (1999). Regulation of cyclooxygenase-2 (COX-2) in rat renal cortex by adrenal glucocorticoids and mineralocorticoids. Proc Natl Acad Sci U S A.

[7] Zhang MZ (2018). Renal medullary interstitial COX-2 (cyclooxygenase-2) is essential in preventing salt-sensitive hypertension and maintaining renal inner medulla/papilla structural integrity. Hypertension.

[8] Harris RC, Zhang MZ (2011). Cyclooxygenase metabolites in the kidney. Compr Physiol.

[9] Harris RC (2004). Cyclooxygenase-2 and the renal renin-angiotensin system. Acta Physiol Scand.

[10] Wang X (2017). Macrophage cyclooxygenase-2 protects against development of diabetic nephropathy. Diabetes.

[11] Nakao S (2005). Infiltration of COX-2-expressing macrophages is a prerequisite for IL-1 beta-induced neovascularization and tumor growth. J Clin Invest.

[12] Kratz M (2014). Metabolic dysfunction drives a mechanistically distinct proinflammatory phenotype in adipose tissue macrophages. Cell Metab.

[13] Coats BR (2017). Metabolically activated adipose tissue macrophages perform detrimental and beneficial functions during diet-induced obesity. Cell Rep.

[14] Tiwari P (2019). Metabolically activated adipose tissue macrophages link obesity to triple-negative breast cancer. J Exp Med.

[15] Wasserman DH (2018). The vasculature in prediabetes. Circ Res.

[16] Byun MR (2013). Phorbaketal A inhibits adipogenic differentiation through the suppression of PPARγ-mediated gene transcription by TAZ. Eur J Pharmacol.

[17] Tsujii M (1998). Cyclooxygenase regulates angiogenesis induced by colon cancer cells. Cell.

[18] Zhang MZ (2015). Inhibition of cyclooxygenase-2 in hematopoietic cells results in salt-sensitive hypertension. J Clin Invest.

[19] Chang J (2015). Prostaglandin E receptor 4 (EP4) promotes colonic tumorigenesis. Oncotarget.

[20] Takayama K (2002). Prostaglandin E2 suppresses chemokine production in human macrophages through the EP4 receptor. J Biol Chem.

[21] Boada-Romero E (2020). The clearance of dead cells by efferocytosis. Nat Rev Mol Cell Biol.

[22] Sachet M (2017). The immune response to secondary necrotic cells. Apoptosis.

[23] Fox CS (2007). Abdominal visceral and subcutaneous adipose tissue compartments: association with metabolic risk factors in the Framingham Heart Study. Circulation.

[24] Pan Y (2022). Myeloid cyclooxygenase-2/prostaglandin E2/E-type prostanoid receptor 4 promotes transcription factor MafB-dependent inflammatory resolution in acute kidney injury. Kidney Int.

[25] Tang EH (2011). Lack of EP4 receptors on bone marrow-derived cells enhances inflammation in atherosclerotic lesions. Cardiovasc Res.

[26] Nataraj C (2001). Receptors for prostaglandin E(2) that regulate cellular immune responses in the mouse. J Clin Invest.

[27] Shi J (2010). The prostaglandin E2 E-prostanoid 4 receptor exerts anti-inflammatory effects in brain innate immunity. J Immunol.

[28] Haka AS (2016). Exocytosis of macrophage lysosomes leads to digestion of apoptotic adipocytes and foam cell formation. J Lipid Res.

[29] Olefsky JM, Glass CK (2010). Macrophages, inflammation, and insulin resistance. Annu Rev Physiol.

[30] Ying W (2019). Expansion of islet-resident macrophages leads to inflammation affecting β cell proliferation and function in obesity. Cell Metab.

[31] Chan PC (2016). Importance of adipocyte cyclooxygenase-2 and prostaglandin E2-prostaglandin E receptor 3 signaling in the development of obesity-induced adipose tissue inflammation and insulin resistance. FASEB J.

[32] Lijnen HR (2008). Rofecoxib impairs adipose tissue development in a murine model of nutritionally induced obesity. Thromb Haemost.

[33] Tian YF (2011). The importance of cyclooxygenase 2-mediated oxidative stress in obesity-induced muscular insulin resistance in high-fat-fed rats. Life Sci.

[34] Ghoshal S (2011). Cyclooxygenase-2 deficiency attenuates adipose tissue differentiation and inflammation in mice. J Biol Chem.

[35] Chan PC (2018). Targeted inhibition of CD74 attenuates adipose COX-2-MIF-mediated M1 macrophage polarization and retards obesity-related adipose tissue inflammation and insulin resistance. Clin Sci (Lond).

[36] Jaworski K (2009). AdPLA ablation increases lipolysis and prevents obesity induced by high-fat feeding or leptin deficiency. Nat Med.

[37] Hu X (2016). Major role of adipocyte prostaglandin E2 in lipolysis-induced macrophage recruitment. J Lipid Res.

[38] Pierre C (2018). Invalidation of microsomal prostaglandin E synthase-1 (mPGES-1) reduces diet-induced low-grade inflammation and adiposity. Front Physiol.

[39] Banhos Danneskiold-Samsoe N (2019). Overexpression of cyclooxygenase-2 in adipocytes reduces fat accumulation in inguinal white adipose tissue and hepatic steatosis in high-fat fed mice. Sci Rep.

[40] Yasui M (2015). The prostaglandin E2 receptor EP4 regulates obesity-related inflammation and insulin sensitivity. PLoS One.

[41] Inazumi T (2020). Prostaglandin E_2_-EP4 axis promotes lipolysis and fibrosis in adipose tissue leading to ectopic fat deposition and insulin resistance. Cell Rep.

[42] Bourlier V (2008). Remodeling phenotype of human subcutaneous adipose tissue macrophages. Circulation.

[43] Wentworth JM (2010). Pro-inflammatory CD11c^+^CD206^+^ adipose tissue macrophages are associated with insulin resistance in human obesity. Diabetes.

[44] Ohinata K, Yoshikawa M (2008). Central prostaglandins in food intake regulation. Nutrition.

[45] Mendes NF (2018). Hypothalamic microglial activation in obesity: a mini-review. Front Neurosci.

[46] Aid S, Bosetti F (2011). Targeting cyclooxygenases-1 and -2 in neuroinflammation: Therapeutic implications. Biochimie.

[47] Schneider A (2004). Generation of a conditional allele of the mouse prostaglandin EP4 receptor. Genesis.

[48] Wang D (2009). Cardiomyocyte cyclooxygenase-2 influences cardiac rhythm and function. Proc Natl Acad Sci U S A.

[49] Ferron M, Vacher J (2005). Targeted expression of Cre recombinase in macrophages and osteoclasts in transgenic mice. Genesis.

[50] Chung S (2018). TGF-β promotes fibrosis after severe acute kidney injury by enhancing renal macrophage infiltration. JCI Insight.

[51] Ayala JE (2011). Hyperinsulinemic-euglycemic clamps in conscious, unrestrained mice. J Vis Exp.

[52] Li Z (2018). Inhibition of epidermal growth factor receptor activation is associated with improved diabetic nephropathy and insulin resistance in type 2 diabetes. Diabetes.

[53] Zhang MZ (2013). Role of epoxyeicosatrienoic acids (EETs) in mediation of dopamine’s effects in the kidney. Am J Physiol Renal Physiol.

