# Supplementary material for: Cyclooxygenase-2 in adipose tissue macrophages limits adipose tissue dysfunction in obese mice
Source: J Clin Invest. 2022 May 2;132(9):e152391. doi: 10.1172/JCI152391 (PMC9057601; doi:10.1172/JCI152391)
Supplement: Supplemental data [file jci-132-152391-s123.pdf]

Supplemental Figures

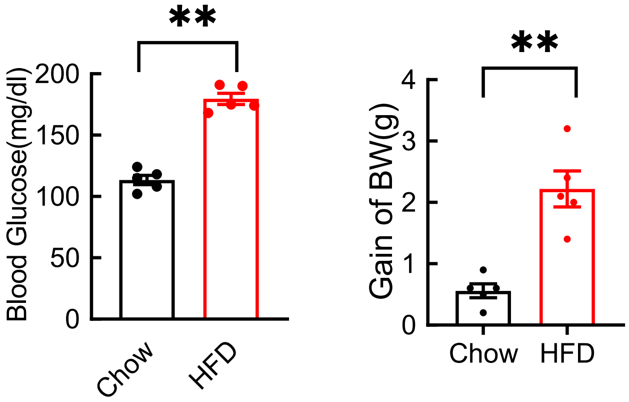

Supplementary Figure S1

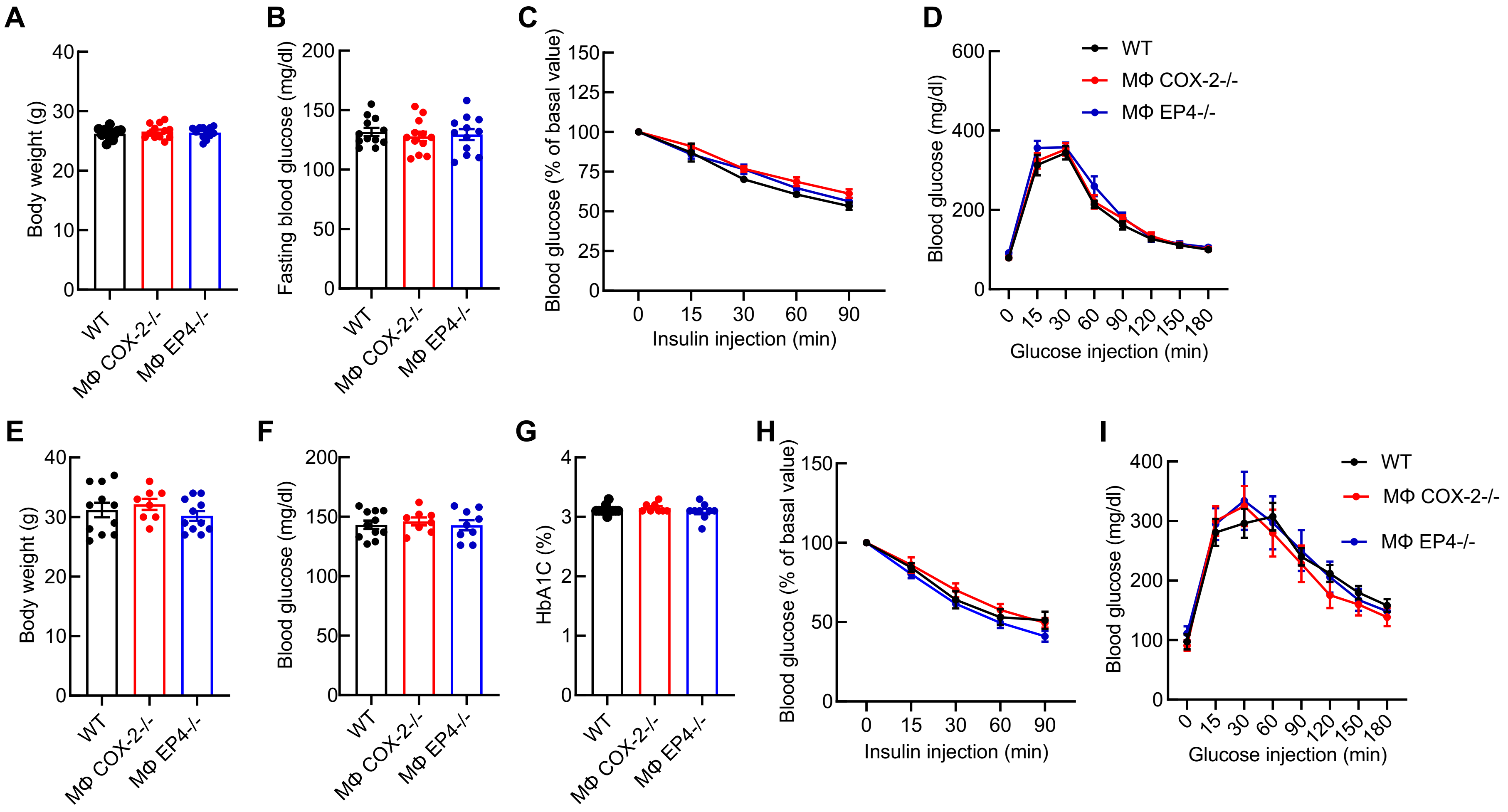

Supplementary Figure S2

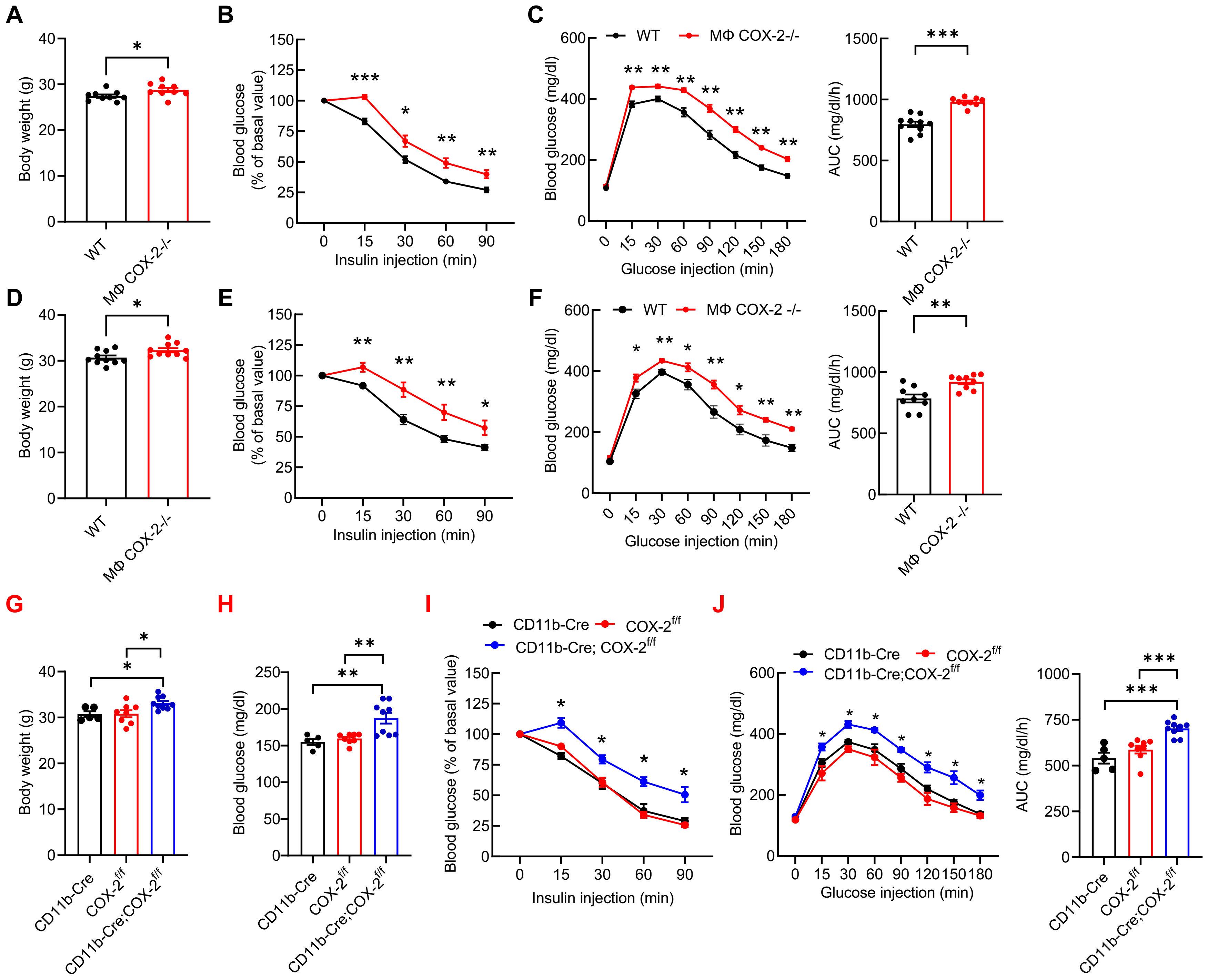

Supplementary Figure S3

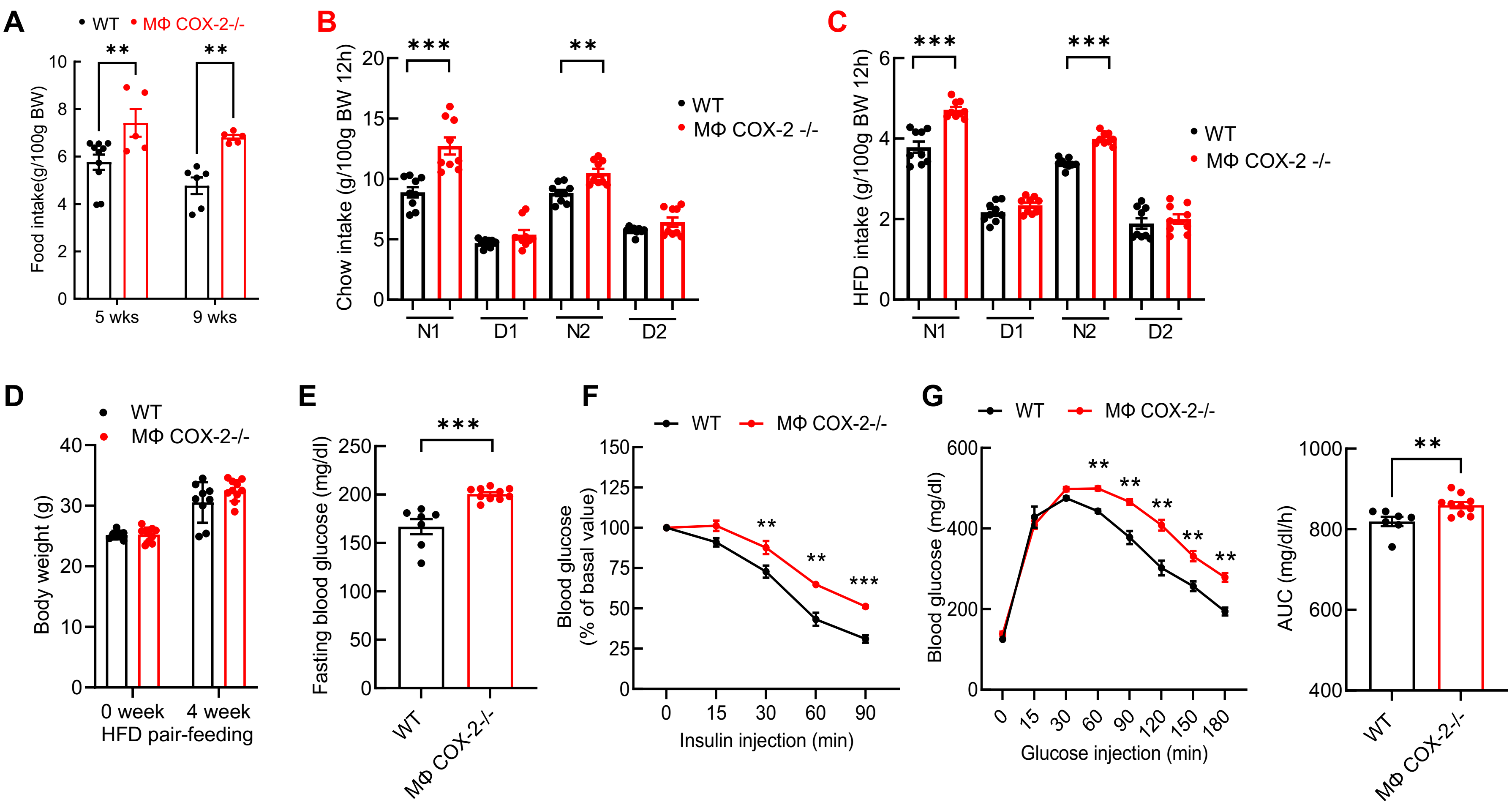

Supplementary Figure S4

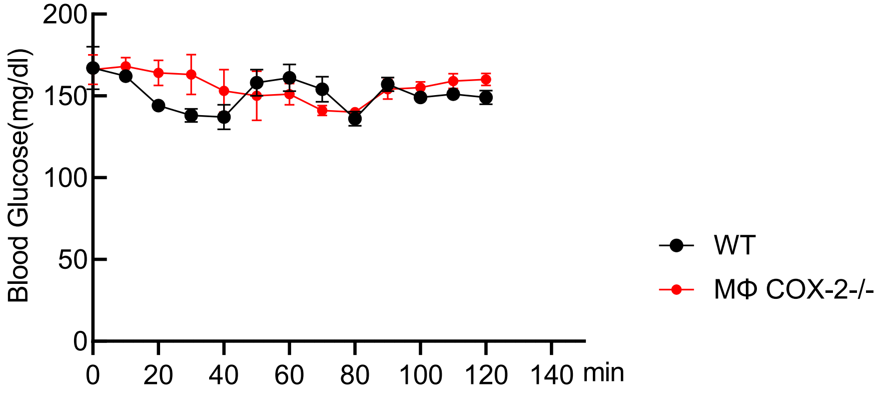

Supplementary Figure S5

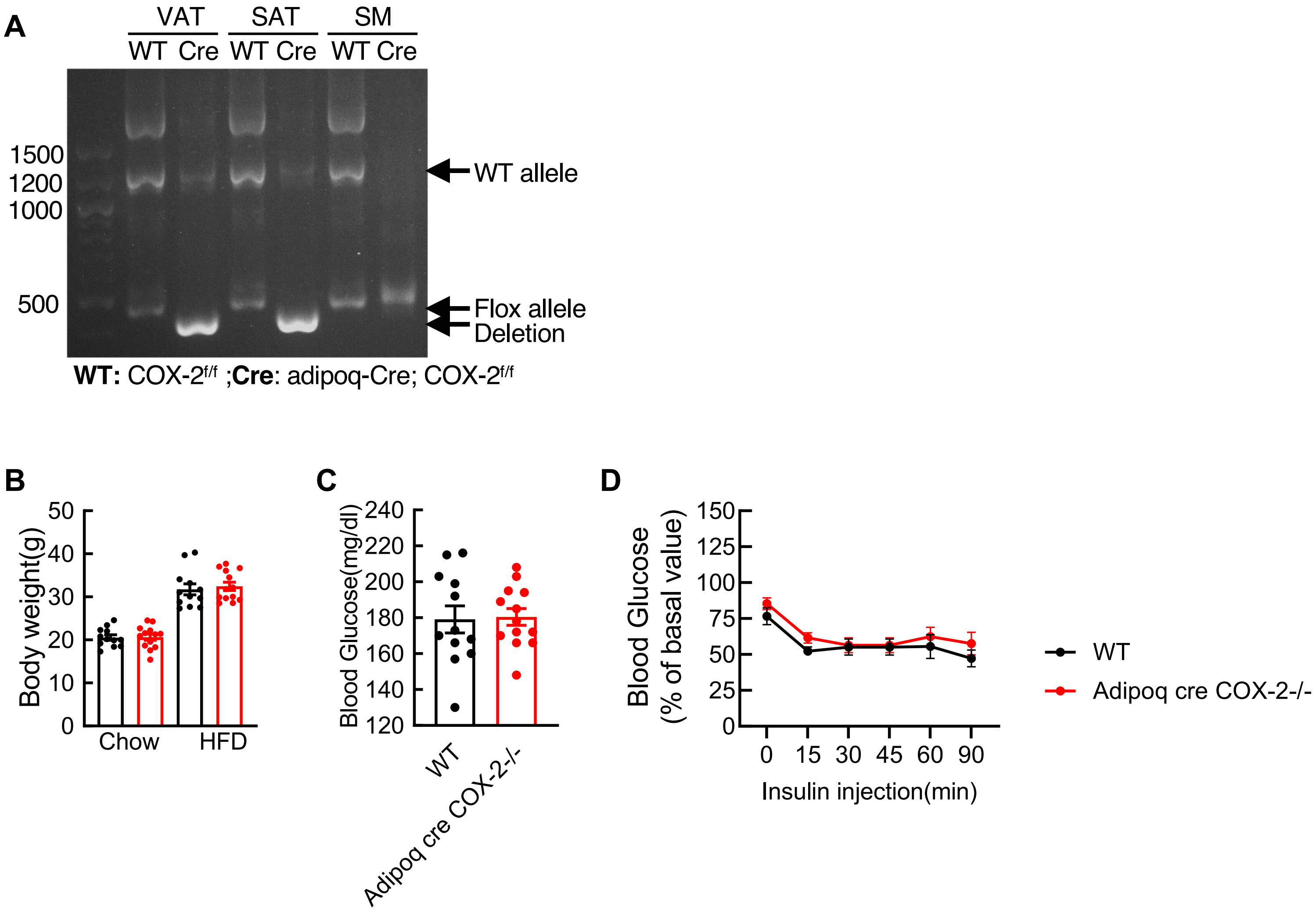

Supplementary Figure S6

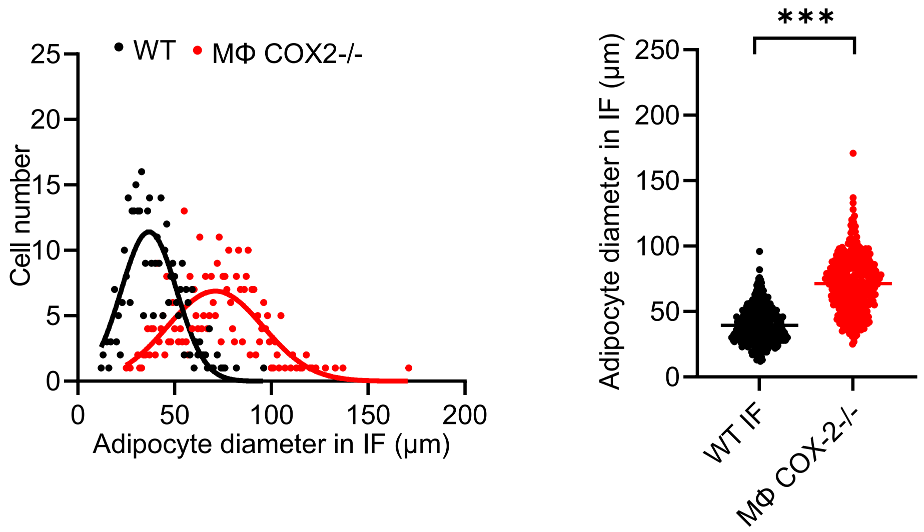

Supplementary Figure S7

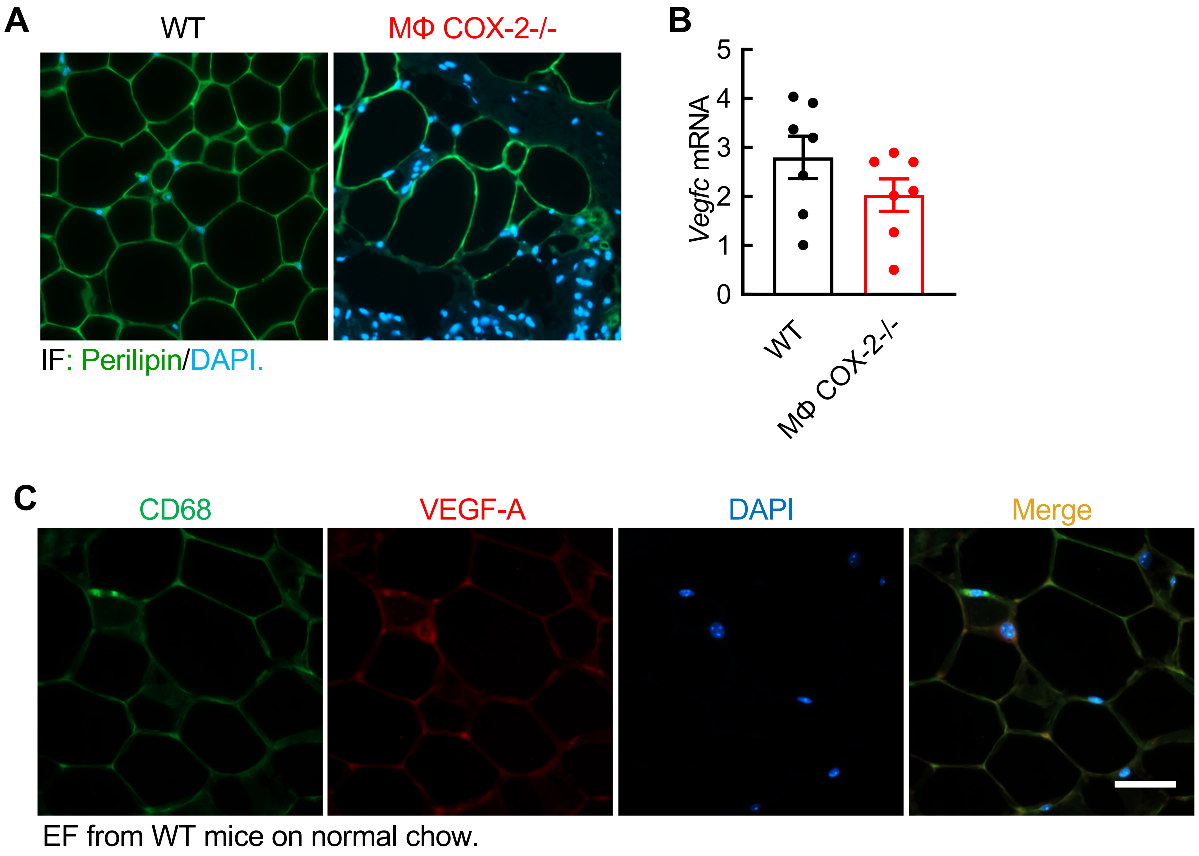

Supplementary Figure S8

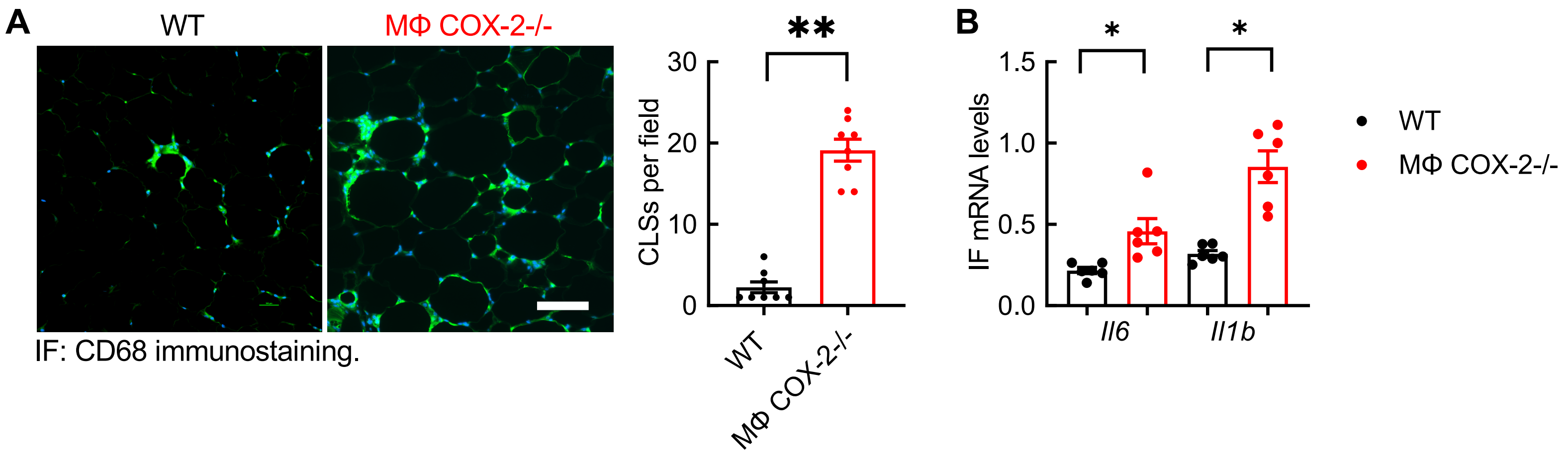

Supplementary Figure S9

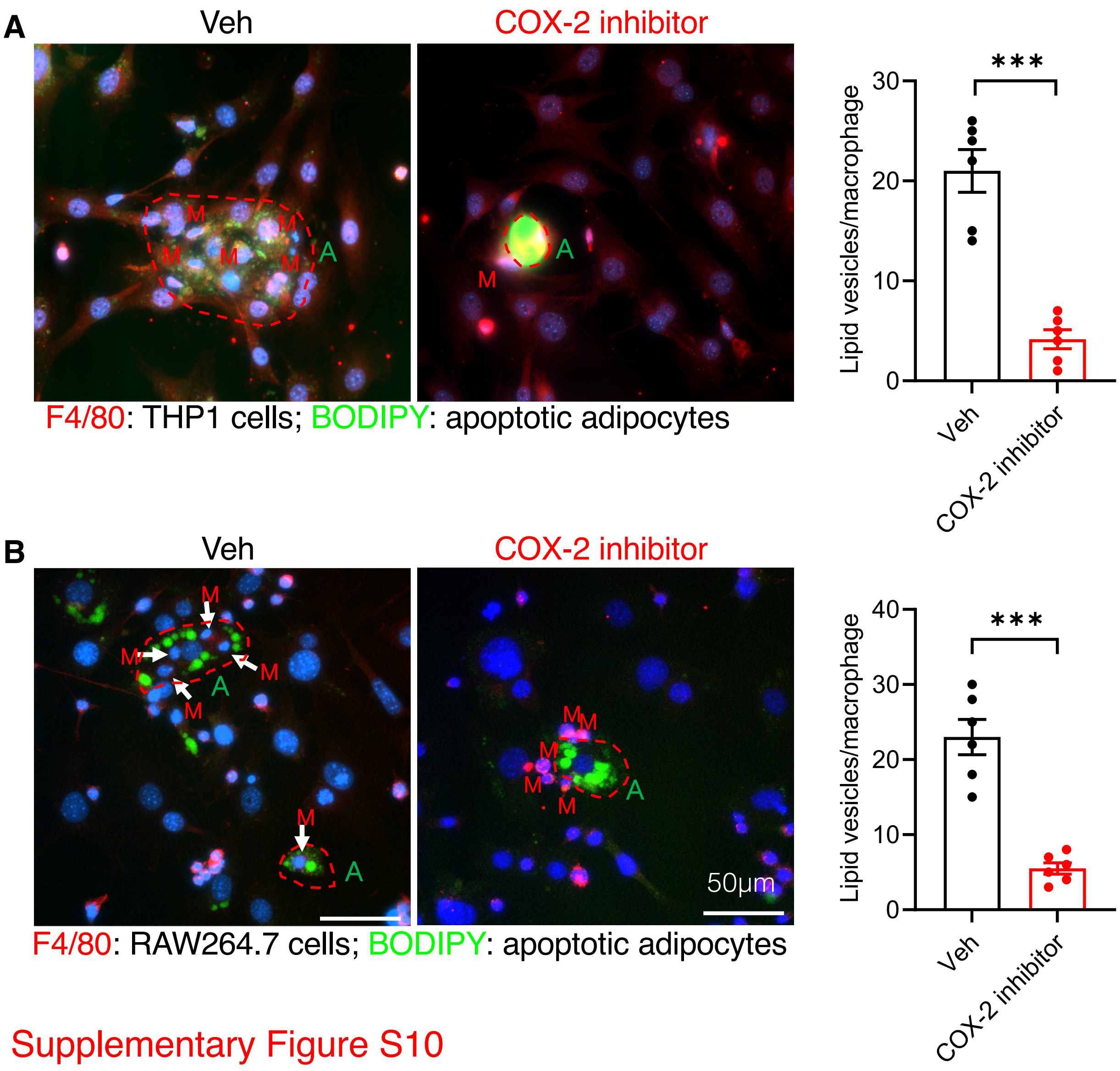

Supplementary Figure S10

Gating strategy for flow cytometry

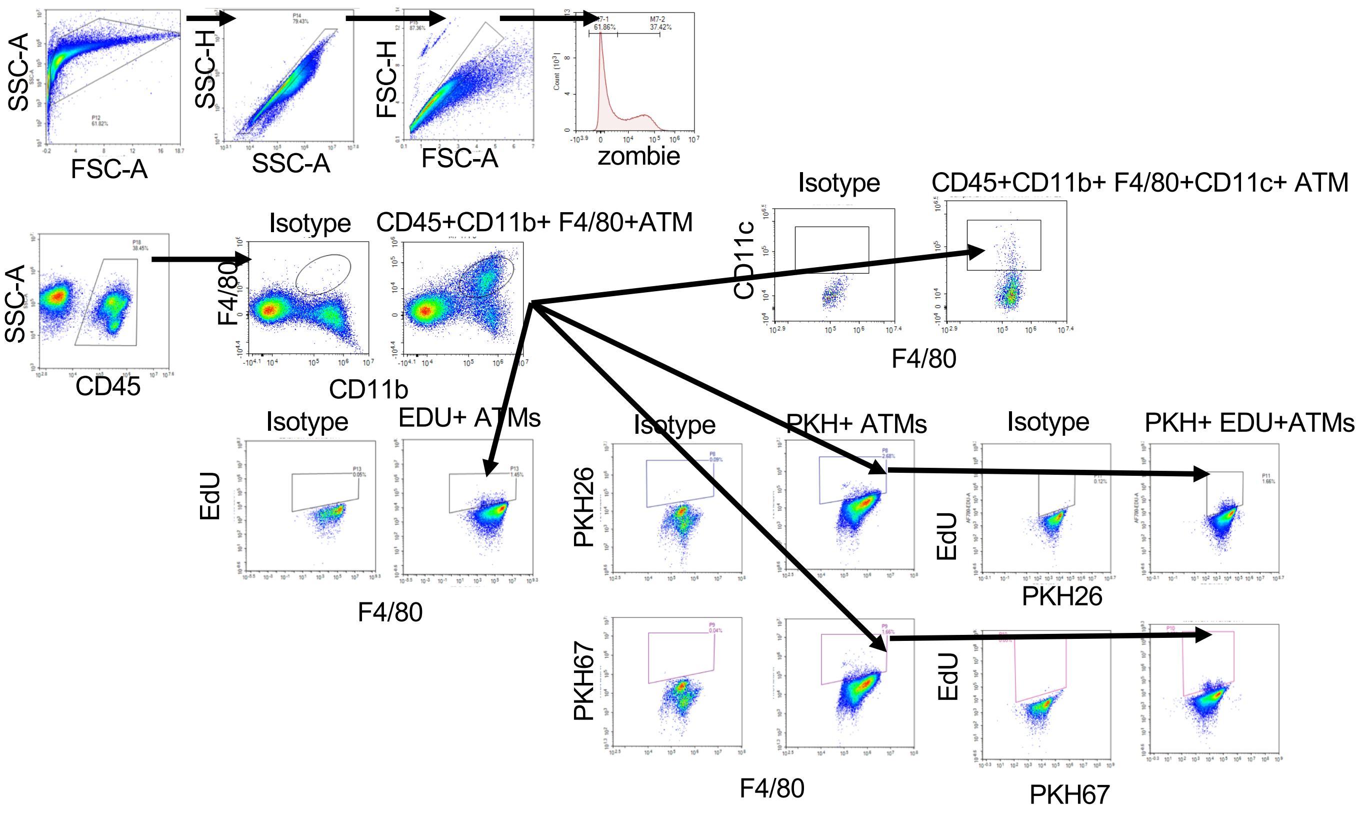

Supplementary Figure S11

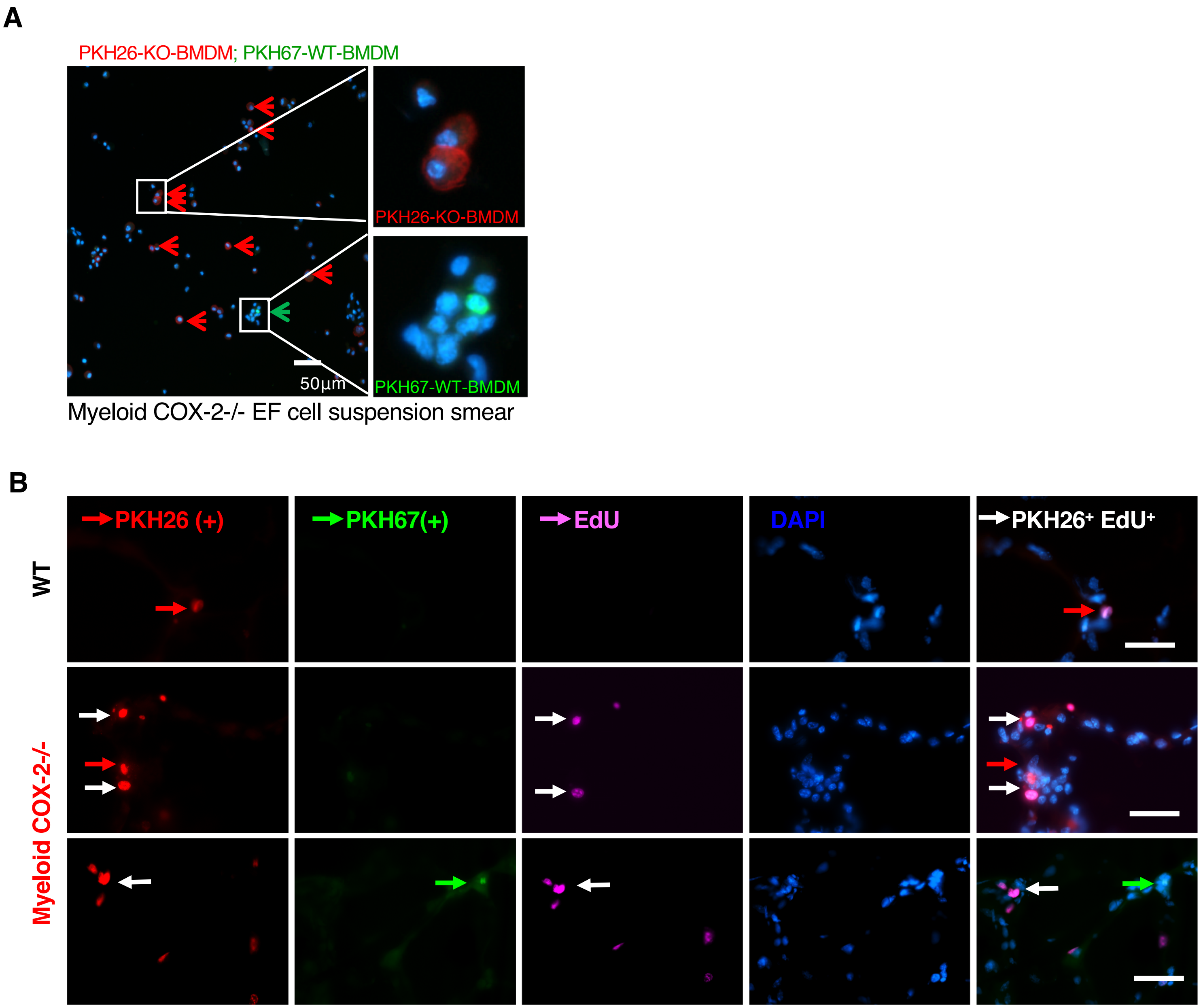

Supplementary Figure S12

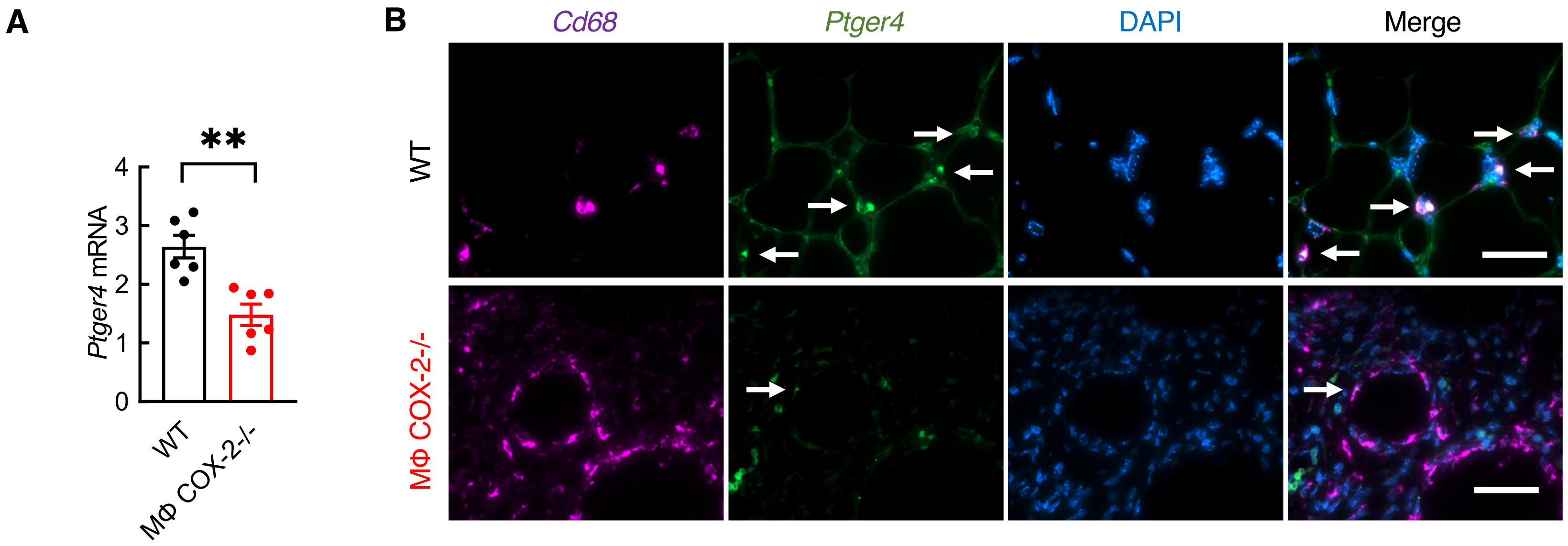

Supplementary Figure S13

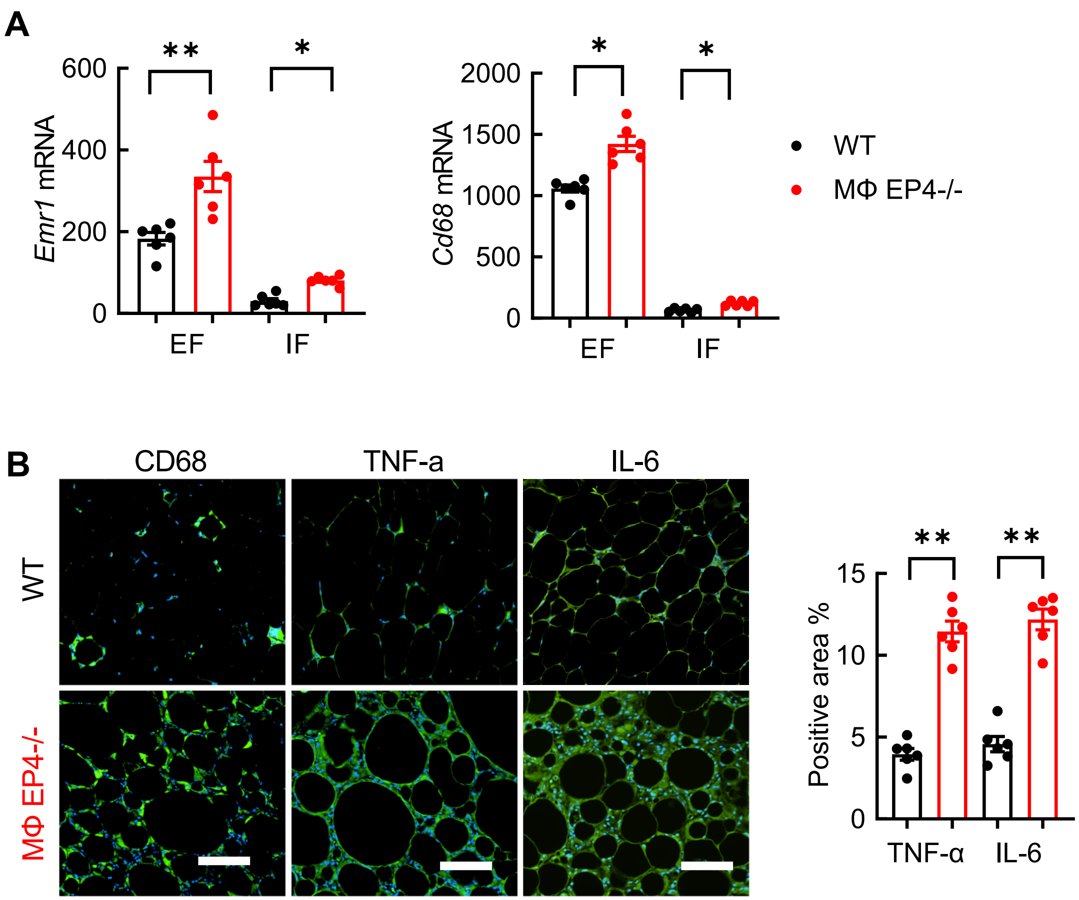

Supplementary Figure S14

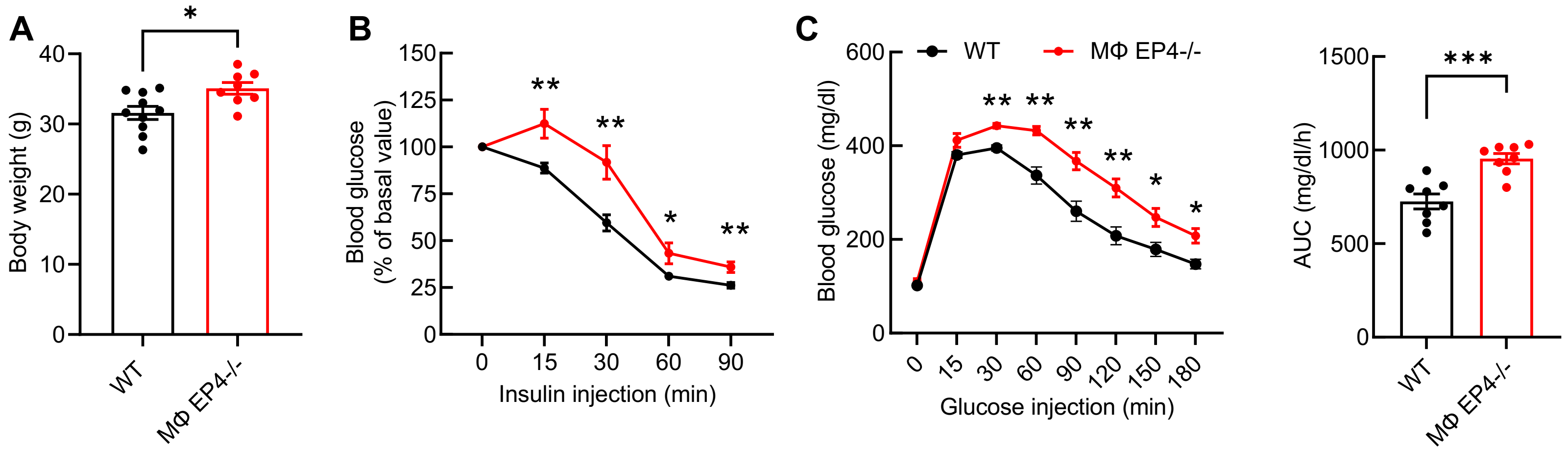

Supplementary Figure S15

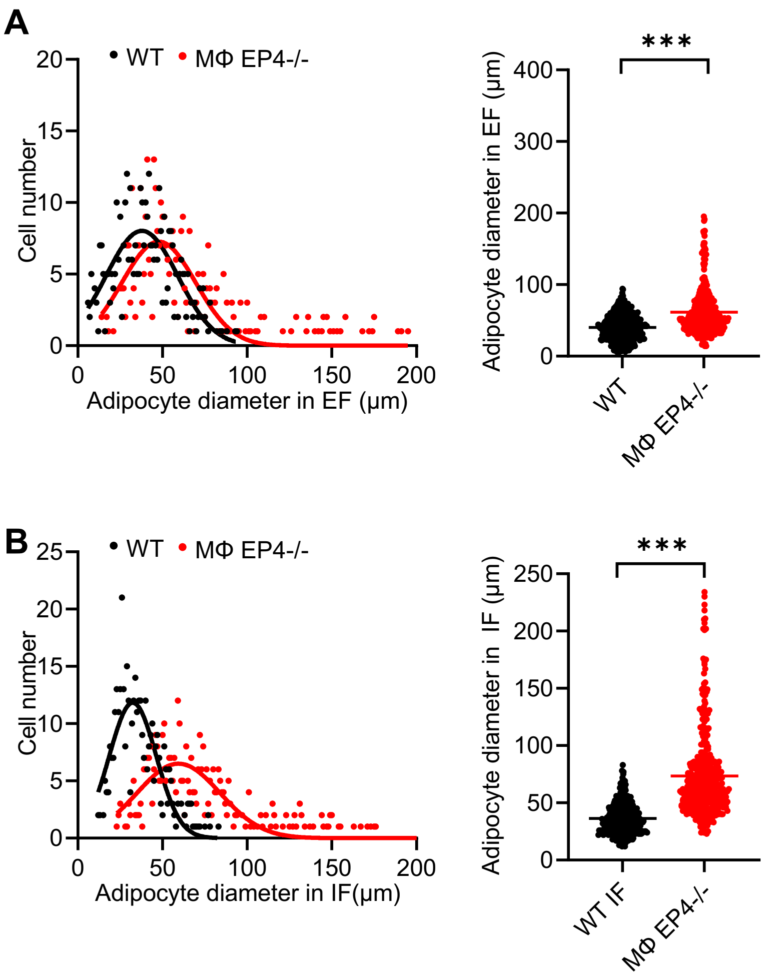

Supplementary Figure S16

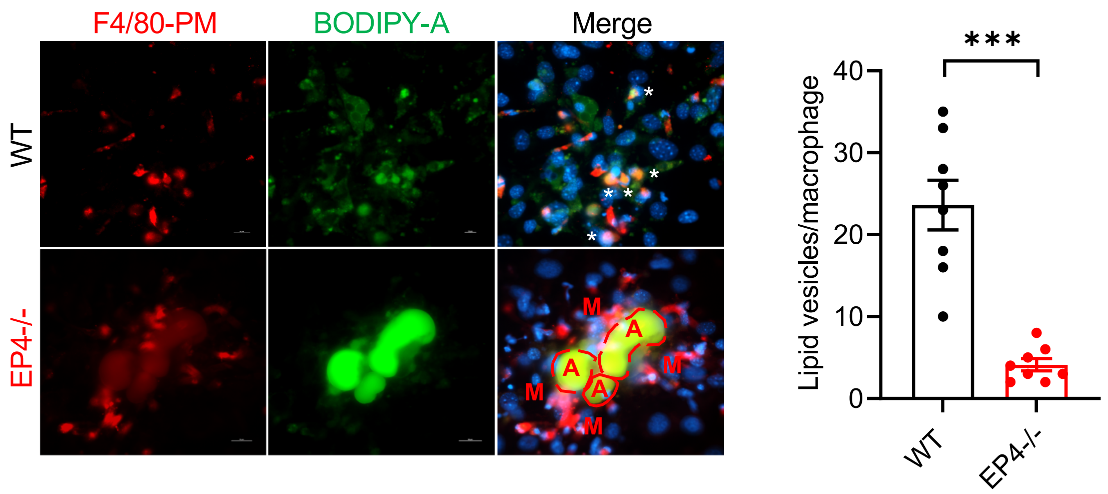

Supplementary Figure S17

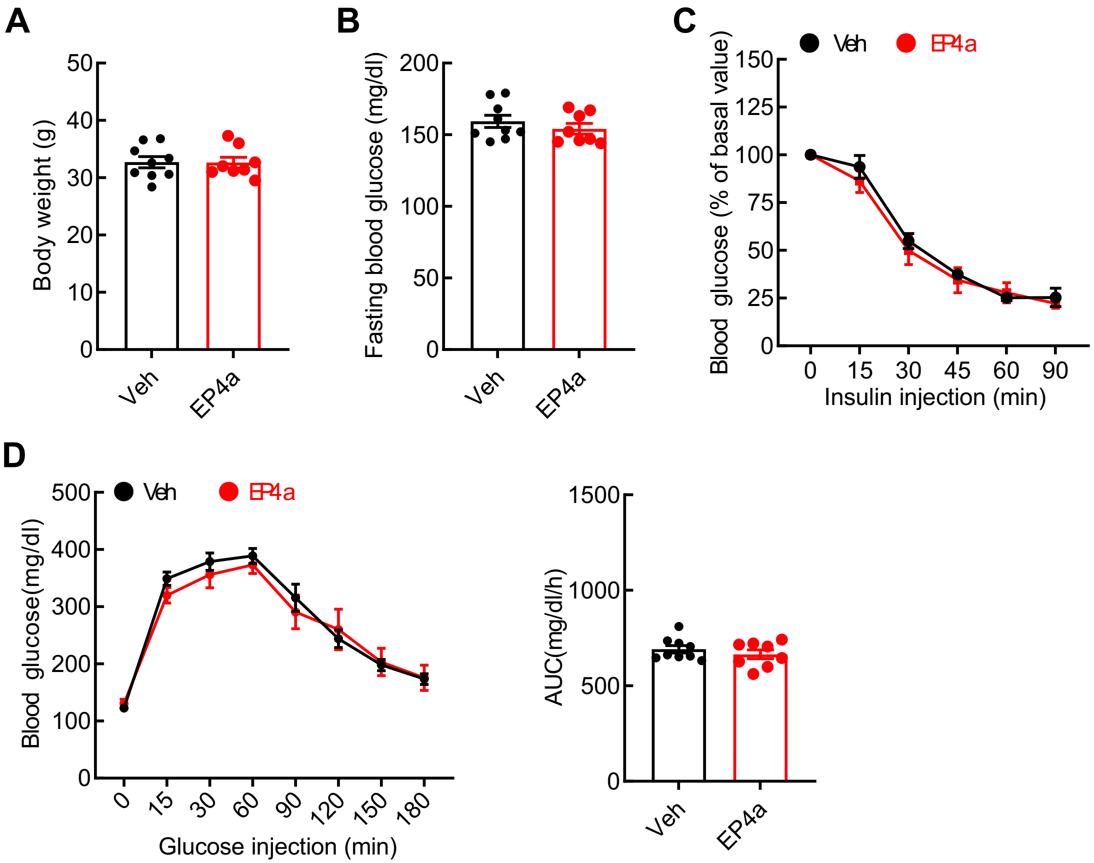

Supplementary Figure S18

Supplementary Figure legend

Figure S1. High fat diet (HFD) for 4 weeks led to increased fasting blood glucose and gain of body weight. N=5.

Data are means ± SEM, ***P*<0.01, analyzed using 2 tailed Student's t test.

Figure S2. Myeloid COX-2-/- mice and myeloid EP4-/- mice had normal metabolic readouts at either 20 or 40 weeks of age on normal chow food. WT (CD11b-Cre) mice, myeloid COX-2-/- (CD11b-Cre; COX-$2^{f/f}$, MΦ COX-2-/-) mice and myeloid EP4-/- mice (CD11b-Cre; $EP4^{f/f}$, MΦ EP4-/-) mice were fed normal chow food for 20 or 40 weeks. (**A**-**D**) Body weight (n=12) (**A**), fasting blood glucose (n=12) (**B**), insulin tolerance (n=5-6) (**C**) and glucose tolerance (n=5-7) (**D**) were comparable among WT, MΦ COX-2-/-, and MΦ EP4-/- mice at 20 weeks of age. (**E**-**I**) Body weight (n=9-11) (**E**), fasting blood glucose (n=9-11) (**F**), HbA1C (n=9-11) (**G**), insulin tolerance (n=5-9) (**H**) and glucose tolerance (n=4-10) (**I**) were also comparable in WT, MΦ COX-2-/-, and MΦ EP4-/- at 40 weeks of age.

Figure S3. Myeloid COX-2-/- mice had more significant metabolic abnormalities 4 weeks after a high fat diet. WT (COX-$2^{f/f}$ and CD11b-Cre) mice and myeloid COX-2-/- (CD11b-Cre; COX-$2^{f/f}$, MΦ COX-2-/-) mice were fed a high fat diet (HFD) or a medium fat diet (MFD) for 4 weeks. (**A**-**C**) Myeloid COX-2-/- mice on a HFD for 4 weeks had greater increases in body weight (n=9) (**A**) as well as decreased insulin tolerance (n=10 or 11) (**B**) and glucose tolerance (n=9 or 10) (**C**). (**D**-**F**) Myeloid COX-2-/- mice on a MFD for 4 weeks also had greater increases in body weight (n=10) (**D**) as well as decreased insulin tolerance (n=9 or 10) (**E**) and glucose tolerance (n=9) (**F**). **(G-J)** CD11b-Cre mice and COX-$2^{f/f}$ had similar body weight, blood glucose, and insulin tolerance and glucose tolerance tests (n=5-9).

Data are means ± SEM, **P*<0.05, ***P*<0.01, ****P*<0.001, analyzed using 2 tailed Student's t test for **A and D,** 2-way ANOVA followed by Tukey's post hoc test for **B, E,** and **I,** 2 tailed Student's t test and 2-way ANOVA followed by Tukey's post hoc test for **C** and **F,** 2-way ANOVA followed by Bonferroni's post hoc test for **G** and **H,** and 2-way ANOVA followed by Bonferroni's post hoc test and 2-way ANOVA followed by Tukey's post hoc test for **J**.

Figure S4. Pair-feeding myeloid COX-2-/- mice led to greater abnormal metabolic readouts in DIO. (**A**) When fed *ad libitum*, MΦ COX-2-/- mice ate more at 5th and 9th week on the HFD (n=5-10).

(**B&C**) Both WT mice and MΦ COX-2-/- mice had the majority of their food intake at night (N) on both chow food (n=9) (**B**) and on HFD (measured after 4 weeks of the diets) (n=9) (**C**). (**D-G**) WT (COX-$2^{f/f}$) mice and myeloid COX-2-/- (CD11b-Cre; COX-$2^{f/f}$, MΦ COX-2-/-) mice were pair fed the high fat diet (HFD) for 4 weeks. Pair-feeding led to similar increases in body weight (n=7-10) (**D**), but higher fasting blood glucose (n=7-10) (**E**) and less insulin tolerance (n=7 or 8) (**F**) and glucose tolerance (n=7-10) (**G**) in MΦ COX-2-/- mice.
Data are means ± SEM, **P*<0.05, ***P*<0.01, ****P*<0.001, analyzed using 2 tailed Student's t test for **A-E,** 2-way ANOVA followed by Tukey's post hoc test for **F,** and 2 tailed Student's t test and 2-way ANOVA followed by Tukey's post hoc test for **G**.

Figure S5. Hyperinsulinemic-euglycemic clamps. WT mice and myeloid COX-2-/- mice were fed the HFD for 11 weeks and hyperinsulinemic-euglycemic clamps were performed. Blood glucose was maintained at constant and similar levels during clamps between myeloid COX-2-/- mice and WT mice. N=4 and 5.

Figure S6. Selective COX-2 deletion in adipocytes had minimal effect on the HFD-induced metabolic abnormalities. Adipoq-Cre; COX-$2^{f/f}$ (Adipocyte COX-2-/-) and COX-$2^{f/f}$ (WT) mice were generated and fed the HFD for 16 weeks. (**A**) Genomic DNAs were isolated from visceral adipose tissue (VAT), subcutaneous adipose tissue (SAT), and skeletal muscle (SM). Deletion of floxed alleles was observed in VAT and SAT but not in SM, an indication of selective COX-2 deletion in adipocytes. (**B**) Body weights were comparable between WT mice and adipocyte COX-2-/- mice fed chow food or the HFD. N=12 and 13. (**C**) Fasting blood glucose levels were comparable between WT mice and adipocyte COX-2-/- mice fed the HFD, n=12 and 13. (**D**) Insulin tolerant test was comparable between WT mice and adipocyte COX-2-/- mice fed the HFD, n=10.

Figure S7. Myeloid COX-2-/- mice had larger adipocytes in inguinal fat (IF) in DIO. WT and myeloid COX-2-/- (CD11b-Cre; COX-$2^{f/f}$, MΦ COX-2-/-) mice were on the HFD for 12 weeks. Myeloid COX-2-/- mice had larger adipocytes in IF. N = 390 (65 from each of 6 mice in each group).
Data are means ± SEM, ****P*<0.001, analyzed using 2 tailed Student's t test.

Figure S8. Myeloid COX-2-/- mice had decreased IF perilipin expression in DIO. WT and myeloid COX-2-/- mice were fed the HFD for 12 weeks. (**A**) Myeloid COX-2-/- mice had decreased IF perilipin expression. Scale bar: 100μm. (**B**) EF *Vegfc* mRNA levels were comparable between WT mice and

myeloid COX-2-/- mice 12 weeks after HFD. N=7. (**C**) VEGF-A expression in EF CD68-positive ATMs was minimal. Scale bar: 50μm.

Figure S9. Myeloid COX-2-/- mice had increased IF ATMs and proinflammatory cytokines in DIO. WT and myeloid COX-2-/- mice were fed the HFD for 12 weeks. (**A**) Myeloid COX-2-/- mice had more IF CLSs. N=8. (**B**) Myeloid COX-2-/- mice had increased inguinal fat (IF) mRNA expression of proinflammatory cytokine *Il6* and *Il1b*. N=6.

Data are means ± SEM, **P*<0.05, ***P*<0.01, analyzed using 2 tailed Student's t test.

Figure S10. COX-2 inhibition impaired macrophage phagocytosis of apoptotic adipocytes *in vitro*. (**A** and **B**) COX-2 inhibition with the selective COX-2 inhibitor (SC236) decreased the ability of the human macrophage-like THP1 cells (**A**) and the mouse macrophage-like RAW264.6 cells (B), to phagocytose apoptotic adipocytes. A: apoptotic adipocytes; M: macrophages; arrows: pointing to macrophages. Scale bar: 50μm.

Data are means ± SEM, ****P*<0.001, analyzed using 2 tailed Student's t test.

Figure S11. Strategy for adipose tissue flow cytometry analysis.

Figure S12. COX-2 deletion led to increased monocyte recruitment and increased ATM proliferation in EF in DIO. WT and myeloid COX-2-/- mice were on the HFD for 4 weeks and EF was used for experiments. (**A**) EF cell suspension smear of myeloid COX-2-/- mice had more PKH26-positive cells (red, COX-2-/- BMDMs) but few PKH67-positive cells (green, WT BMDMs), indicating more infiltrated COX-2-/- BMDMs. (**B**) Proliferation of infiltrated PKH26 labeled COX-2-/- BMDMs ($PKH26^{+}EdU^{+}$) was only observed in EF from myeloid COX-2-/- recipients. No proliferation of infiltrated PKH67 labeled WT BMDMs ($PKH67^{+}EdU^{+}$) was observed. Red arrows: $PKH26^{+}$ COX-2-/- BMDMs; white arrows: proliferating $PKH26^{+}EdU^{+}$COX-2-/- BMDMs; green arrow: $PKH67^{+}$ WT BMDM. Scale bar: 50μm.

Figure S13. Myeloid COX-2-/- mice had decreased EF *Ptger4* mRNA expression in ATMs in DIO. WT and myeloid COX-2-/- mice were on the HFD for 12 weeks. (**A**) EF *Ptger4* mRNA levels were lower in MΦ COX-2-/- mice than in WT mice. N = 6. (**B**) RNAscope determined that EF *Ptger4* mRNA and *Cd68* mRNA double positive cells (arrows) were decreased in myeloid COX-2-/- mice. Arrows: *Ptger4* expression ATMs. Scale bar: 50μm.

Data are means ± SEM, ***P*<0.01, analyzed using 2 tailed Student's t test.

Figure S14. **Myeloid EP4-/- mice had increased EF ATM accumulation and proinflammatory cytokines in DIO.** WT (EP4$^{f/f}$) and myeloid EP4-/- (CD11b-Cre; EP4$^{f/}$f; MΦ EP4-/-) mice were on the HFD for 12 weeks. (**A**) Myeloid EP4-/- mice had higher *Emr1* and *Cd68* expression in EF and IF. N=6. (**B**) Myeloid EP4-/- mice had increased EF CD68+ ATMs and proinflammatory cytokines (TNF-□ and IL-6). N=6. Scale bar: 100μm.

Data are means ± SEM, **P*<0.05, ***P*<0.01, analyzed using 2-way ANOVA followed by Bonferroni's post hoc test for **A**.

Figure S15. Myeloid EP4-/- mice had more significant metabolic abnormalities 4 weeks after DIO. WT (EP4$^{f/f}$) mice and myeloid EP4-/- (CD11b-Cre; EP4$^{f/f}$, MΦ EP4-/-) mice were fed the high fat diet (HFD) for 4 weeks. (**A-C**) Myeloid EP4-/- mice had greater increases in body weight (n=8 or 10) (**A**) as well as decreased insulin tolerance (n=8) (**B**) and glucose tolerance (n=8 or 10) (**C**).

Data are means ± SEM, **P*<0.05, ***P*<0.01, analyzed using 2 tailed Student's t test for **A,** 2-way ANOVA followed by Tukey's post hoc test for **B,** and 2 tailed Student's t test and 2-way ANOVA followed by Tukey's post hoc test for **C**.

Figure S16. Myeloid EP-/- mice had larger adipocytes in epididymal fat (EF) and inguinal fat (IF) in DIO. WT and myeloid EP4-/- (CD11b-Cre; EP4$^{f/f}$, MΦ EP4-/-) mice were on the HFD for 12 weeks. Myeloid EP4-/- mice had larger adipocytes in both EF (**A**) and IF (**B**). N = 390 (65 from each of 6 mice in each group).

Data are means ± SEM, ****P*<0.001, analyzed using 2 tailed Student's t test.

Figure S17. EP4 deficiency impaired macrophage phagocytosis of apoptotic adipocytes *in vitro*. Peritoneal macrophages (PMs) were isolated from WT and MΦ EP4-/- mice 3 days after peritoneal thioglycolate injection. Phagocytosis of apoptotic adipocytes (green, A) by WT peritoneal macrophages (PMs, red, F4/80), as indicated by PMs containing adipocyte-derived lipid (asterisks), was impaired in EP4-/- PMs isolated from myeloid EP4-/- mice. N=8. Scale bar: 50μm.

Data are means ± SEM, ****P*<0.001, analyzed using 2 tailed Student's t test.

Figure S18. An EP4 agonist had minimal effect on the HFD-induced metabolic abnormalities in WT mice. WT mice were treated with the HFD for 10 weeks with or without the selective EP4 agonist (EP4a, ONO-4819), given via minipump at a dose of 75 μg/kg/day throughout the 10-week experimental period). (**A-D**) The EP4a had minimal effect on the HFD-induced gain of body weight (**A**), fasting blood glucose (**B**), insulin tolerance (**C**) or glucose tolerance (**D**). N=8 or 9.
